# CD83, a Novel MAPK Signaling Pathway Interactor, Determines Ovarian Cancer Cell Fate

**DOI:** 10.3390/cancers12082269

**Published:** 2020-08-13

**Authors:** Aalia Batool, Hao Liu, Yi-Xun Liu, Su-Ren Chen

**Affiliations:** 1 Laboratory of Cell Proliferation & Regulation Biology, College of Life Sciences, Beijing Normal University, Beijing 100875, China; aaliabatool@sina.com (A.B.); liuhaobnu89@yeah.net (H.L.); 2 Laboratory of Reproductive Neuroendocrinology, Department of Zoology, Faculty of Biological Sciences, Quaid-i-Azam University, Islamabad 45320, Pakistan; 3 State Key Laboratory of Stem Cell and Reproductive Biology, Institute of Zoology, Chinese Academy of Sciences, Beijing 100101, China; liuyixun37@21cn.com

**Keywords:** ovarian cancer, proliferation, stemness, CD83, MAPK signaling pathway, MAP3K7, FOXO1, STAT3

## Abstract

Ovarian cancer is a leading cause of death from gynecologic malignancies worldwide. Although CD83 is widely described as a solid marker for mature dendritic cells, emerging pieces of evidence indicate the expression of membrane protein CD83 by various tumor cells, including ovarian cancer cells. However, the potential role of CD83 in ovarian cancer cell properties and development remains absolutely unknown. By using human CD83 stable overexpression and knockdown sublines of several ovarian cancer cells, we observed that CD83 advanced the growth proliferation, colony formation ability, spheroid formation, and in vivo tumorigenicity of ovarian cancer cells; surprisingly, CD83 limited their migration and invasion potentials. Positive regulation of proliferation/stemness factors (e.g., cyclin-CDKs and KIT/CD44) but negative regulation of matrix metallopeptidases (e.g., MMP1 and 7) by CD83 were revealed by the integrated analysis of transcriptome and proteome. Furthermore, immunoprecipitation-mass spectrometry (IP-MS) and co-immunoprecipitation (Co-IP) first identified the association of CD83 with MAP3K7 (also known as TAK1) and MAP3K7-binding protein TAB1 on the cell membrane. Moreover, CD83 functions through the activation of MAP3K7-MEK1/2-ERK1/2 cascades to further regulate downstream FOXO1/p21/CDK2/CCNB1 and STAT3/DKK1 signaling pathways, thus activating proliferation and spheroid formation of ovarian cancer cells, respectively. Collectively, our findings define a CD83-MAPK pathway in the regulation of proliferation and stemness in ovarian cancer cells, with potential therapeutic applications in blocking their progression.

## 1. Introduction

Ovarian cancer is the most lethal of the gynecologic malignancies [1]. The majority of cases are discovered when the primary tumor has already metastasized, and the 5-year survival rate of these patients reduces to <20% [2]. Moreover, the recurrent disease occurs in approximately 75% of patients with advanced ovarian cancer within 3 years after surgery and chemotherapy [3]. In ovarian cancer, malignant cells are derived from primary tumors as single cells and/or spheroids, and diffuse multifocal intraperitoneal metastasis and malignant ascites are representative characteristics.

Multiple signaling pathways have been investigated to be involved in the regulation of ovarian cancer cell progression. Among them is the mitogen-activated protein kinase (MAPK) cascade, which regulates the proliferation, migration, invasion, and chemotherapy resistance of ovarian cancer cells [4,5,6,7]. MAPK signaling pathway is activated at the cell membrane by a number of extra- and intracellular stimuli. The phosphorylated and activated MAPKKKs subsequently phosphorylate MAPKKs, which, once activated, phosphorylate MAPKs. FOXO family of transcription factors are characterized by a forkhead DNA binding domain. FOXOs are well-known as tumor suppressors and induce cell cycle arrest by increasing the expression of p21/p27 and decreasing the expression of cyclins/CDKs [8,9]. In ovarian cancers, FOXOs are involved in cell cycle progression, cell survival, and chemosensitivity [10,11]. Recent studies have defined the critical role of the STAT3-DKK1 signaling pathway in the generation of cancer stem cells in a variety of tumors, including ovarian cancer [12,13,14]. However, the regulators and molecular mechanisms underlying MAPK activation and downstream signaling transduction in ovarian cancer cell fate are not fully understood.

CD83 encodes a 40–45 kDa surface glycoprotein in the immunoglobulin superfamily and has been widely described as a solid marker for mature dendritic cells (DCs) [15,16]. Tumor-infiltrating CD83-positive DCs are of great importance in initiating the primary antitumor immune response and are confirmed as an independent, immunologic prognostic parameter for survival in patients with various types of cancers [17,18,19,20]. Further literature suggests a wider expression and broader functional role, including in the regulation of B cell function [21] and CD4-positive T cells [22,23]. Soluble CD83, the extracellular domain of the membrane-bound CD83, is released by CD83-positive cells, such as mature DCs, activated T cells, B cells, and natural killer cells [24,25,26,27,28], and could induce tolerogenic DCs and block T cell proliferation [25,28,29,30,31]. Soluble CD83 has been found in the plasma of patients with chronic lymphocytic leukemia and breast cancer [32,33,34] and synovial fluid of early-stage rheumatoid arthritis patients [35]. Interestingly, CD83 is also expressed by tumor cells from lung cancer [24,29,36], cervical cancer [37], and Hodgkin lymphoma [31], and soluble CD83 has been found to be secreted by those tumor cells [24,31,38]. Novel pieces of evidence in favor of the notion that CD83 is an emerging therapeutic target for cancer cells are as follows: (i) melanoma cells with enforced expression of CD83 induce antitumor immunity [39]; (ii) anti-human CD83 antibody with toxin monomethyl auristatin E conjugate kills CD83-positive Hodgkin lymphoma tumor cells [31].

Considering the expression of transmembrane CD83 in various tumor cells exhibiting potential therapeutic implications, this study was conducted with an aim to investigate the role of membrane protein CD83 in ovarian cancer cells. An integrated analysis of proteomic/transcriptomic data and immunoprecipitation-mass spectrometry (IP-MS) were utilized to explore CD83 downstream targets and membrane protein CD83-interacting partners. In this study, we found that CD83 associated with MAP3K7 and activated MAPK cascades to further regulate FOXO1/p21/CDK2/CCNB1 and STAT3/DKK1 signaling pathways, thus activating proliferation and spheroid formation of ovarian cancer cells.

## 2. Results

### 2.1. CD83 Expression Profile in Human Ovarian Cancer Cells and Tissues

**Figure 1:**
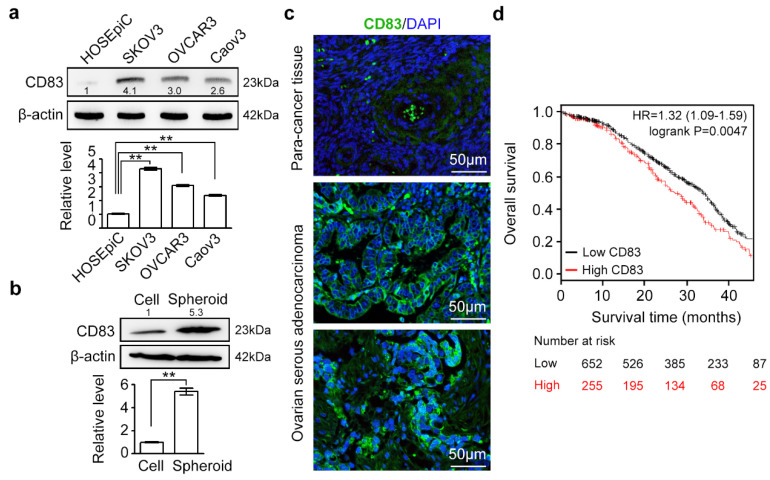
CD83 was ectopically activated in ovarian cancer cells/tissues, and its high expression predicted decreased survival rates. (**a**) Protein level of CD83 in human ovarian surface epithelial cell HOSEpiC and various ovarian serous adenocarcinoma cell lines (e.g., SKOV3, OVCAR3, and Caov3). PNGase F (NEB P0704S) was utilized to remove all N-linked oligosaccharides from glycoproteins. (**b**) The protein level of CD83 in SKOV3 cells and SKOV3 single cell-derived spheroids. The data in a and b were presented as the mean±SEM; Student’s *t*-test; ** *p* < 0.01. (**c**) Expression and membrane distribution of CD83 (Abcam, ab205343) in ovarian serous adenocarcinoma and para-cancer tissue samples (OriGene, CT565862) were revealed by immunofluorescent staining. Scale bar, 50 μm. (**d**) Kaplan–Meier analysis of overall survival based on CD83 levels in 907 ovarian cancer patients (low CD83 expression: 652, the high expression: 255) from the The Cancer Genome Atlas (TCGA) database. High expression of CD83 was closely relative to the reduced overall survival rates (*p* = 0.0047). The uncropped western blots of Figure 1a,b in the Figures S8 and S9.

To obtain the expression information of CD83 in human ovarian cancer, we first re-analyzed gene expression microarray data of GSE105437 [40] and found that *CD83* level was higher (logFC > 2, *p* < 0.01) in high-grade serous ovarian cancers (HGSOC) (*n* = 10) than that in normal ovary tissues (*n* = 5). Second, CD83 expression was significantly upregulated in ovarian serous adenocarcinoma cell lines (e.g., SKOV3, OVCAR3, and Caov3) as compared with ovarian surface epithelial cell HOSEpiC (Figure 1a). Spheroids, enriched for cancer stem cells, serve as the vehicle for transcoelomic metastasis and growth of ovarian cancer [41,42]. Interestingly, CD83 was found to be hyperactivated in ovarian cancer spheroids derived from a single cancer cell (Figure 1b). Immunostaining analysis further indicated the ectopically activated CD83 expression in human ovarian serous adenocarcinoma, as compared with that in para-cancer tissues (Figure 1c). Among ovarian cancer cell subpopulations, *CD83* transcript was significantly upregulated (logFC >2, *p* < 0.01) in EpCAM^+^CD45^+^ highly aggressive, drug-resistant, and ovarian cancer stem cell-containing tumor cells compared to EpCAM^+^ cells, as revealed by GSE75036 [43] (Figure S1). Besides *CD83*, a large number of the *CD83* highly-associated genes (Person correlation >0.5, *p* < 0.01) within ovarian cancer transcriptome showed higher expression in ovarian cancer cells, especially in the EpCAM^+^/CD45^+^ subtypes (Figure S1). *CD83* highly-associated genes, such as *ITGAM*, *IL1B*, *CYBB*, *PIK3R5*, *BTK,* and *OSM*, ectopically express in ovarian cancer cells or tissues, involving in ovarian cancer progression, hypoxia networks, and the development of chemoresistance [44,45,46,47,48,49]. Finally, we found that ovarian cancer patients’ survival inversely correlated with the level of *CD83* expression in 907 ovarian cancer patients (low CD83 expression: 652, the high expression: 255), as patients with high *CD83* expression lived significantly shorter compared with their counterparts with low CD83 expression (Figure 1d). Taken together, *CD83* was associated with poor survival of ovarian cancer patients, and CD83 might determine the fate of ovarian cancer cells.

### 2.2. CD83 Advances Growth Proliferation, Spheroid Formation, and In Vivo Tumorigenic Capacity of Ovarian Cancer Cells

**Figure 2:**
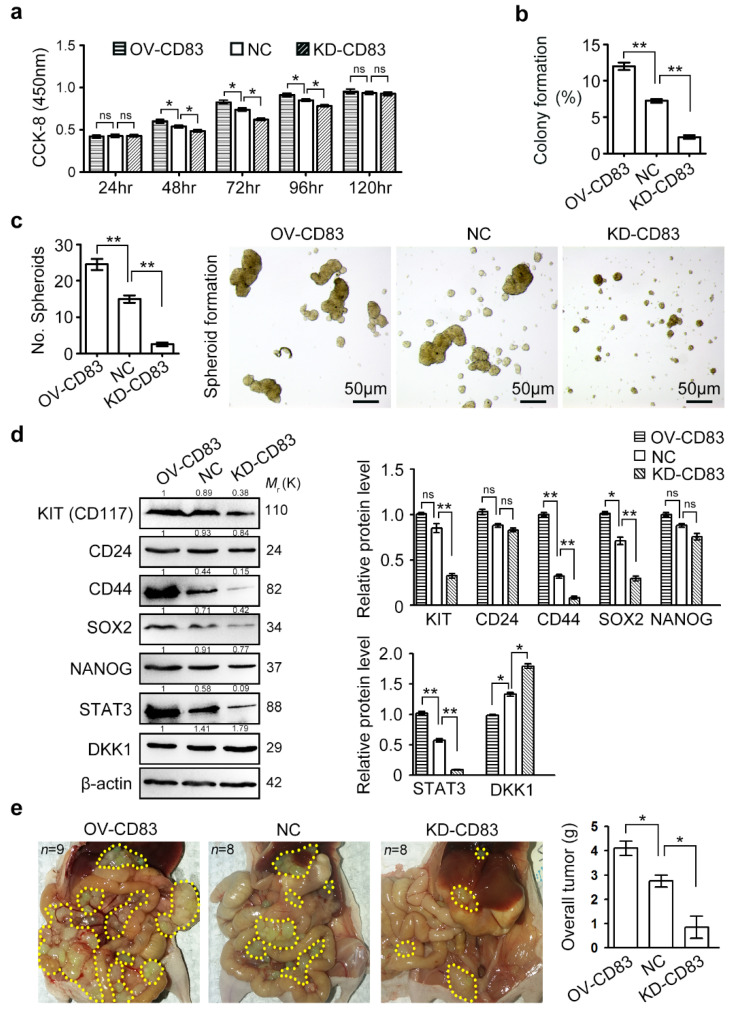
CD83 promoted growth proliferation, spheroid formation, and in vivo tumor growth of ovarian cancer cells. (**a**) SKOV3 cell proliferation was significantly attenuated by CD83 knockdown, whereas CD83 overexpression significantly stimulated the proliferation, as revealed by Cell Counting Kit-8 (CCK-8) assay. (**b**) Colony formation of SKOV3 cells (500 cells per 60 mm dish) was significantly promoted by enforced expression of CD83. (**c**) The number of spheroids derived from SKOV3 cells, which are cultured onto ultra-low attachment plates in cancer stem cell culture medium. Scale bar, 50 μm. (**d**) Expression of stemness factors, STAT3, and DKK1 identified by Western blotting. The uncropped western blots of Figure 2d in the Figure S10. (**e**) Intraperitoneal injection of SKOV3 cells (1 × 10^6^) into nude mice. The total tumor weights for CD83-OV groups (*n* = 9) were significantly higher than that for the control groups (*n* = 8) and CD83-KD groups (*n* = 8). The differences between NC and OV, as well as NC and KD, were presented as the mean±SEM; Student’s *t*-test; * *p* < 0.05, ** *p* < 0.01, ‘ns’ means not significant.

To study the pathological functions of CD83, we generated human CD83 stable overexpression (CD83-OV) and knockdown (CD83-KD) sublines of multiple ovarian cancer cells (e.g., SKOV3, OVCAR3, and Caov3), using infection of full-length CD83 lentivirus and triple CD83-specific lentiviral shRNA lentivirus, respectively. GFP-positive stable transfected cell lines were generated by subsequently two rounds of puromycin selection. qRT-PCR and ELISA assay showed that CD83 expression in SKOV3 cells was significantly upregulated after overexpression, whereas both the mRNA and protein levels of CD83 were significantly reduced in CD83-KD cancer cells (Figure S2). We first examined the roles of CD83 on the main characteristics of ovarian cancer cells, including their proliferation, stemness, apoptosis, migration, and invasion abilities. Compared with the control group, the proliferation behavior of SKOV3 cells was significantly attenuated in the CD83-KD group, whereas the accelerated proliferation of CD83-OV cells was observed (Figure 2a). Consistent with the CCK-8 assay, a colony formation assay further showed that CD83 expression level, indeed, determined the colony formation ability of single SKOV3, OVCAR3, and Caov3 cells (Figure 2b, Figure S3a,b). On the other hand, apoptosis rates of CD83-KD, CD83-OV, and control SKOV3 cells were almost identical in vitro, as revealed by a TUNEL staining (Figure S4).

Spheroids are enriched for cancer stem cells and serve as the vehicle for transcoelomic metastasis and growth of ovarian cancer [31,32,41,42]. Hyperactivation of CD83 in ovarian cancer spheroids (Figure 1b) indicated that CD83 might be indispensable in the regulation of spheroid formation. To test this hypothesis, cultures of CD83-KD, CD83-OV, and control ovarian cancer cells were dissociated, and single cells were then plated on ultra-low attachment culture plates with stem cell medium to allow compact spheroid formation for 7 days. Notably, CD83 knockdown significantly reduced the spheroid formation ability of the single SKOV3, OVCAR3, and Caov3 cells, while forced expression of CD83 boosted the growth of spheroids (Figure 2c, Figure S3c,d). Compared with CD83-OV and control groups, CD83-KD cancer cells showed a reduction of stemness factors, including KIT, CD44, and SOX2 (Figure 2d, Figure S5a,b). Chen et al. defined that a STAT3-miR-92a-DKK1 network regulated spheroid formation in ovarian cancer [12]. Consistent with this observation, the downregulated STAT3 and the elevated DKK1 were observed in CD83-KD SKOV3 cells (Figure 2d).

To determine the relevance of these findings in vivo, we subsequently examined the effect of CD83 on tumorigenicity in nude mice receiving an intraperitoneal injection of manipulated SKOV3 cells. The total tumor weights for CD83-OV groups (*n* = 9) were significantly higher than that for the control (*n* = 8) and CD83-KD groups (*n* = 8), suggesting that the expression of CD83 was tightly linked to tumor burden (Figure 2e). Taken together, these data indicated the importance of the CD83 in the promotion of the growth proliferation and spheroid formation in vitro, as well as in vivo tumor growth.

### 2.3. CD83 Limits Invasion and Migration Ability of Ovarian Cancer Cells

**Figure 3:**
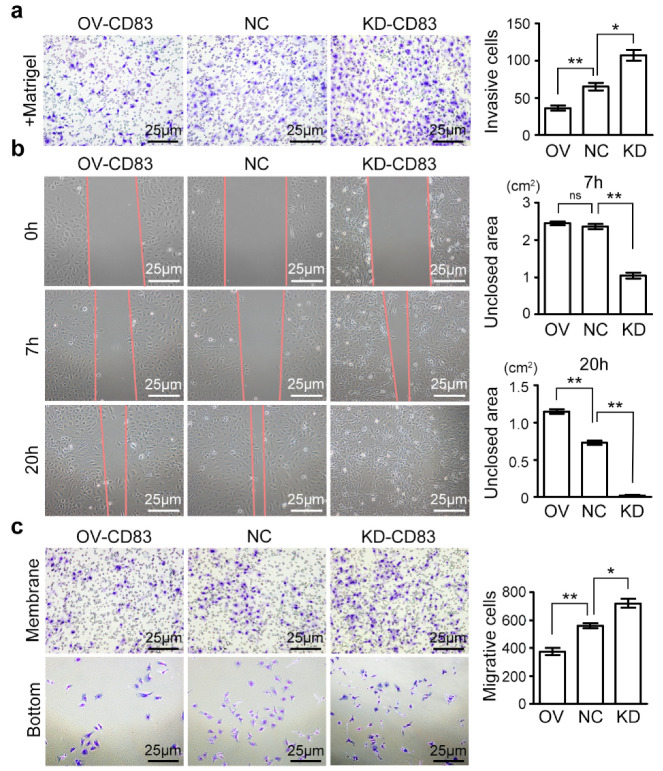
CD83 inhibited the invasion and migration of ovarian cancer cells. (**a**) Representative images and quantification of invasive SKOV3 cells in OV-CD83, NC, and KD-CD83 transwells using matrigel invasion assay. Scale bar, 25 μm. (**b**) The migration ability of SKOV3 cells was determined by wound-healing assay, and the closed wound area was calculated at 0, 7, and 20 h. (**c**) Representative images and quantification of migrative cells using a transwell migration assay. Scale bar, 25 μm. The differences between NC and OV, as well as NC and KD, were presented as the mean ± SEM; Student’s *t*-test; * *p* < 0.05, ** *p* < 0.01, ‘ns’ means not significant.

The invasive capacity was measured via the transwell matrigel invasion assay. We found that enforced CD83 expression in SKOV3, OVCAR3, and Caov3 cells significantly reduced their invasive capabilities to invade the matrigel-embedded transwell, whereas knockdown of CD83 stimulated the invasion of ovarian cancer cells (Figure 3a, Figure S3e,f). The migration ability of SKOV3 cells was determined by the wound-healing assay, and the closed wound area was calculated at 0, 7, and 20 h (Figure 3b). The migration of SKOV3 cells was further measured by the quantification of migrative cells using the transwell migration assay (Figure 3c). Both the wound-healing assay and transwell migration assay indicated that CD83 negatively regulated the migration of SKOV3 cells.

### 2.4. Positive Regulation of Proliferation/Stemness Factors but Negative Regulation of Matrix Metallopeptidases by CD83

**Figure 4:**
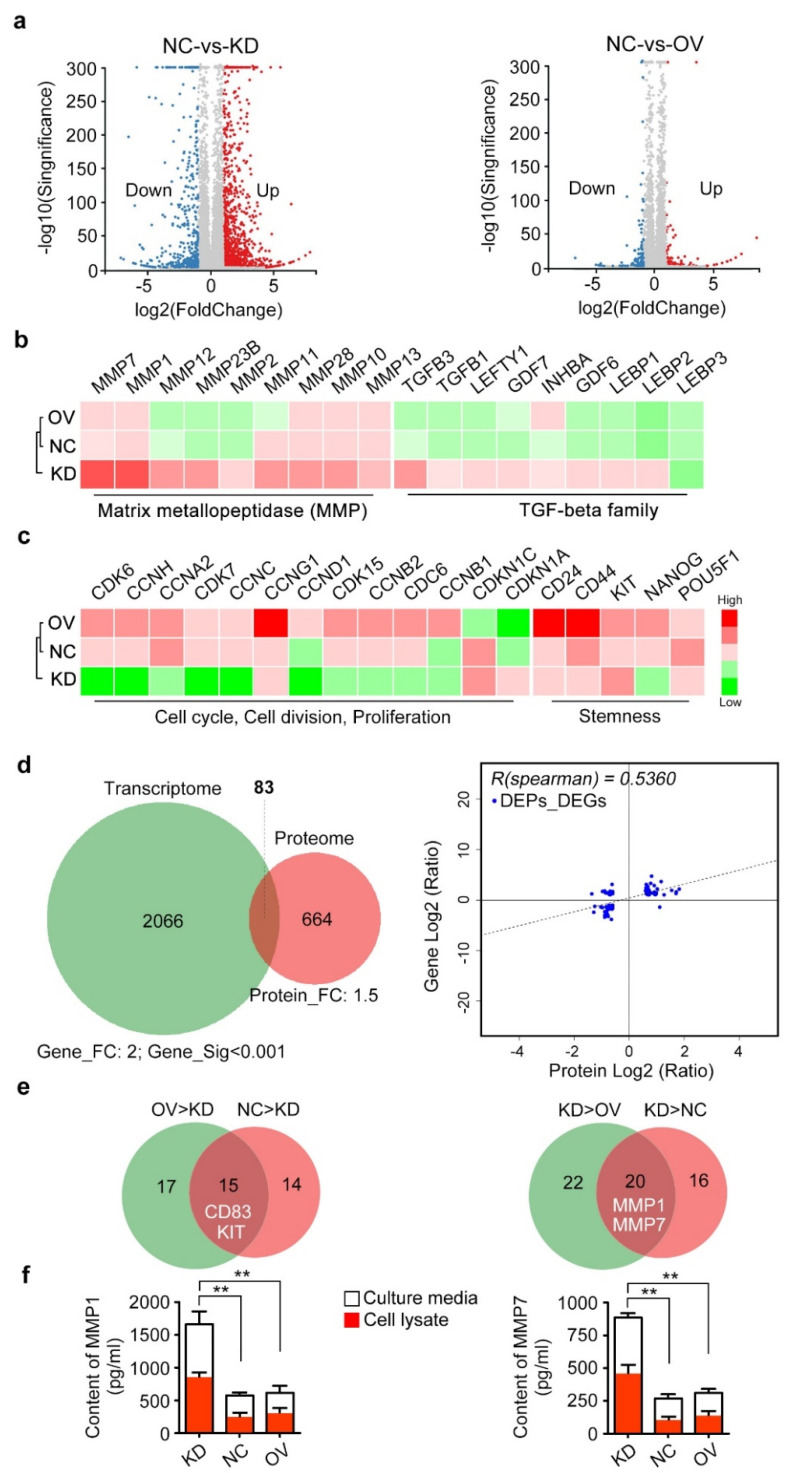
Integrated transcriptome and proteome analyses. (**a**) Volcano plots show the differentially expressed genes of NC-vs.-KD and NC-vs.-OV, as revealed by transcriptome. (**b**) CD83-KD SKOV3 cells exhibited elevated expression of matrix metallopeptidases (MMPs) and TGF-beta family members as compared with NC and CD83-OV cells. (**c**) The expression of cell cycle regulators and stemness-related genes was upregulated in CD83-OV SKOV3 cells. (**d**) The intersection of the transcriptome (fold change ≥2) and proteome (fold change ≥1.5) identified 83 DE proteins (and protein-coding genes) between CD83-KD and NC SKOV3 cells. Correlation analysis of DE genes and DE proteins indicated the consistency (R = 0.5360) between transcriptome and proteome in this study. (**e**) Venn diagram shows the downregulated proteins (e.g., CD83, KIT) and upregulated proteins (e.g., MMP1, MMP7) in the CD83-KD SKOV3 cells. (**f**) Content of MMP1 and MMP7 in SKOV3 cell lysate and culture media by ELISA assay. The differences between KD and NC, as well as KD and OV, were presented as the mean±SEM; Student’s *t*-test; ** *p* < 0.01.

To explore the downstream targets and regulatory network of transmembrane CD83 in ovarian cancer cells without bias, an integrated study of transcriptome and proteome was performed using CD83-OV, CD83-KD, and control SKOV3 cells (Figure S6, Tables S1–4). A great difference between CD83-KD ovarian cancer cells and the other two groups was obviously observed (Figure 4a). We found that CD83-KD ovarian cancer cells exhibited elevated transcriptional expression of matrix metallopeptidases (MMPs) and transforming growth factor (TGF)-beta family members (Figure 4b); in contrast, cell cycle regulators (e.g., cyclins-CDKs) and stemness factors (e.g., CD24, CD44, and KIT) were upregulated in CD83-OV and negative control (NC) ovarian cancer cells (Figure 4c). The intersection of the transcriptome (fold change ≥2) and proteome (fold change ≥1.5) included 83 differentially-expressed (DE) targets between CD83-KD and NC ovarian cancer cells, whereas OV-vs.-NC showed only two DE targets (CD83 and RSPH4A) (Figure 4d). Correlation analysis of DE genes and DE proteins indicated the consistency (R = 0.5360) between transcriptome and proteome in this study. Consistent with this transcriptomic observation, proteomics identified that KIT was attenuated, whereas MMP1 and MMP7 were elevated in CD83-KD ovarian cancer cells (Figure 4e). In addition to MMPs, expression of a disintegrin and metallopeptidases (ADAMs), ADAM metallopeptidase with thrombospondin type 1 motifs (ADAMTSs), and tissue inhibitor of metallopeptidases (TIMPs) also showed differential expression between CD83-KD and other two groups (Figure S7). Enzyme-linked immunosorbent assay (ELISA) further showed the significantly increased contents of MMP1 and MMP7 in both cell lysate and culture media of CD83-KD SKOV3 cells (Figure 4f, Figure S5b–f). Taken together, transcriptome and proteome strongly indicated the positive regulation of proliferation/stemness factors and negative regulation of matrix metallopeptidases by CD83, partially explaining why CD83 advanced the growth proliferation and spheroid formation but limited the migration and invasion of ovarian cancer cells.

### 2.5. CD83 Functions through Its Association with MAP3K7 and Activation of MAPK Signaling Pathway

**Figure 5:**
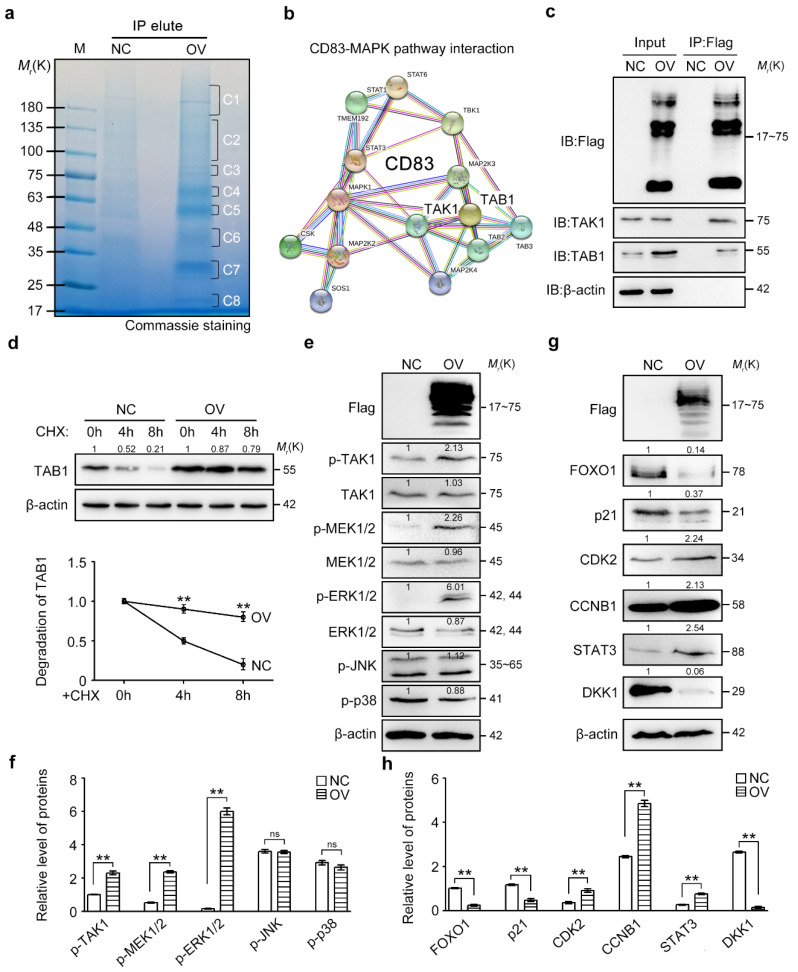
Transmembrane CD83 interacted with TAK1 and TAB1 and activated MAPK and downstream signaling pathways. (**a**) CD83 C-terminal 3×Flag lentiviral plasmid and the stable cell lines were constructed. Membrane CD83 was successfully pulled-down and eluted by a 3×Flag tag, as indicated by Western blotting. The extracts isolated using 3×Flag-target were separated by SDS-PAGE and visualized using Coomassie brilliant blue staining. Eight components of CD83 immunoprecipitated products were excised for the following in-gel trypsin digestion. (**b**) Notably, several members of MAPK family, such as TAK1, TAB1, TAB2, TAB3, MAP2K4, MAPK1, MAP2K2, and MAP2K3, were identified in CD83 pull-down complexes as the main fraction by LC-MS/MS analysis. (**c**) A Co-IP assay in SKOV3 cells was performed to verify the interaction of TAK1 and TAB1 with CD83. (**d**) Degradation of TAB1 in control and OV-CD83 SKOV3 cells treated with 25 μg/mL cycloheximide (CHX), a blocker of the translation, for the indicated time. (**e**,**f**) Expression of members belonging to the MAPK signaling pathway, including p-TAK1, TAK1, p-MEK1/2, MEK1/2, p-ERK1/2, ERK1/2, p-JNK, and p-p38, between control and OV-CD83 SKOV3 cells. (**g**,**h**) Expression analysis of FOXO1, p21, CDK2, Cyclin B1, STAT3, and DKK1 between control and OV-CD83 SKOV3 cells. The differences between NC and OV were presented as the mean±SEM; Student’s *t*-test; ** *p* < 0.01, ‘ns’ means not significant. The uncropped western blots of Figure 5c–e,g in the Figure S11–14.

To dissect the molecular mechanisms of CD83 in the fate determination of ovarian cancer cells, we used the immunoprecipitation, followed by mass spectrometry (IP-MS) approach, to identify protein candidates that functionally associate with transmembrane CD83 (Figure 5a). The details of IP-MS identification are illustrated in Table S5. Notably, several components of mitogen-activated protein kinase (MAPK) family, such as mitogen-activated protein kinase kinase kinase 7 (MAP3K7) (also known as TAK1), TGF-beta activated kinase 1 (MAP3K7) binding proteins (TAB1, TAB2, and TAB3), MAP2K4, MAPK1, MAP2K2, and MAP2K3, were identified in CD83 pull-down complexes as the main fraction (Figure 5b). MAPK signaling pathway acts as an integration point for multiple biochemical signals, involving in a wide variety of cellular processes, such as proliferation and differentiation [50]. MAP3K7 (TAK1), a MAPKKK, forms a kinase complex, including TAB1, TAB2, and TAB3, to active downstream MAP2K4 and MAPK8 [51]. Given that TAK1 and TAB1 are preassociated on the cell membrane, as well as the membrane localization of CD83, we hypothesized that CD83 forms a complex with TAK1-TAB1 to activate the MAPK signaling pathway. We explored the nature of the interaction between CD83 and TAK1-TAB1 in SKOV3 cells by co-immunoprecipitation (Co-IP) experiments and confirmed the CD83-TAK1 and CD83-TAB1 interactions (Figure 5c). Interestingly, CD83 further promoted the stability of TAB1 when SKOV3 cells were treated by cycloheximide, a protein synthesis inhibitor (Figure 5d). Upon CD83 overexpression in SKOV3 cells, the protein levels of phosphorylated (p-)TAK1, p-MEK1/2, and p-ERK1/2 were significantly increased, whereas their total levels remained unchanged, indicating the activation of a MAPK-MEK-ERK signaling pathway (Figure 5e,f). As known, the p-ERK1/2-FOXO1-p21 signaling pathway controls the cyclins/CDKs-mediated cancer cell proliferation [52,53,54], and the STAT3-DKK1 signaling pathway regulates spheroid formation of ovarian cancer cells [12]. Next, we examined the involvement of p-ERK1/2-FOXO1-p21 and STAT3-DKK1 signaling pathways in the regulation of proliferation and stemness of ovarian cancer cells by the newly-identified CD83-TAK1-TAB1 complex. In CD83-OV SKOV3 cells, the expression of the transcription factor FOXO1 and cell cycle progression inhibitor p21 was significantly attenuated, whereas CDK2 and cyclin B1 levels were elevated as compared with that in control groups (Figure 5g,h). On the other hand, the elevated STAT3 expression and downregulation of DKK1 were observed in CD83-OV SKOV3 cells as compared with control groups (Figure 5g,h). Taken together, these data suggested that CD83 formed a complex with TAK1-TAB1 on the cell membrane to further activate downstream p-ERK1/2-FOXO1-p21-CDK2 and STAT3-DKK1 signaling pathways in ovarian cancer cells.

**Figure 6:**
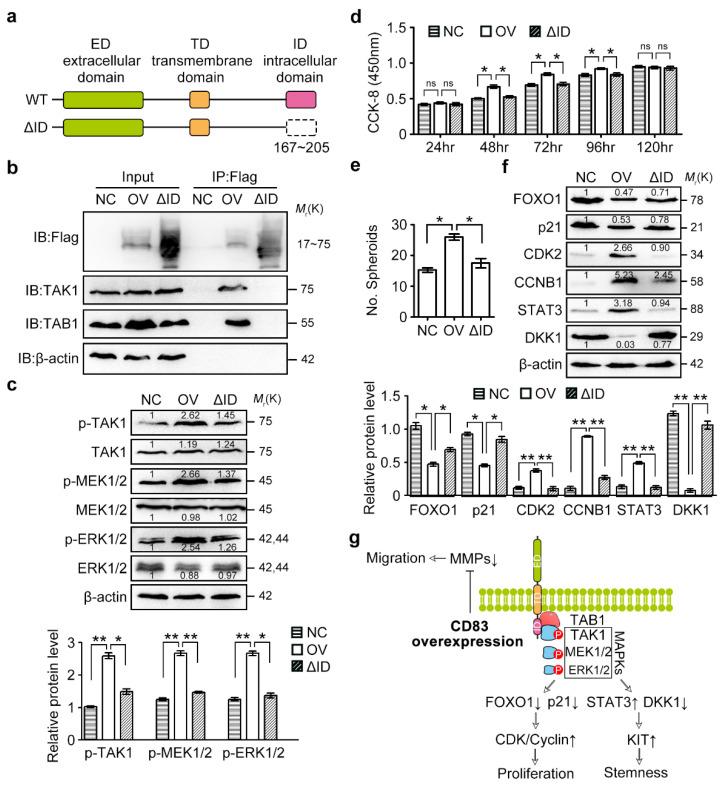
CD83 functioned through its intracellular domain that associated with the TAK1/TAB1 complex. (**a**) Schematic representation of CD83 protein and the truncated mutant. CD83 protein includes the extracellular domain (ED), the transmembrane domain (TD), and the intracellular domain (ID). The CD83 mutant without the ID was named as CD83ΔID. (**b**) Co-IP was performed after the transfection of CD83ΔID with a Flag tag in SKOV3 cells. (**c**) Western blotting detection of the expression of members belonging to the MAPK signaling pathway, including p-TAK1, TAK1, p-MEK1/2, MEK1/2, p-ERK1/2, and ERK1/2. (**d**) In vitro proliferative ability of SKOV3 cells after transfection of wild-type CD83 or CD83ΔID was examined by CCK-8 assay. (**e**) Spheroid formation assay was performed in SKOV3 cells transfected with wild-type CD83 or CD83ΔID. (**f**) Expression analysis of FOXO1, p21, CDK2, Cyclin B1, STAT3, and DKK1 after expression of wild-type CD83 or CD83ΔID in SKOV3 cells. The differences between NC and OV, as well as NC and ΔID, were presented as the mean±SEM, Student’s *t*-test; * *p* < 0.05, ** *p* < 0.01, ‘ns’ means not significant. (**g**) Schematic representation of the biologic roles of CD83 in ovarian carcinogenesis. Transmembrane CD83 directly interacted with MAP3K7 (TAK1) and its partner TAB1 via its intercellular domain (ID). Thus, CD83 acted as an activator of ovarian cancer cell proliferation and stemness by targeting the MAPK signaling pathway and regulating downstream FOXO1/p21/Cyclin/CDK and STAT3/DKK1 cascades. In the graphical abstract, ectopic activation of membrane CD83 activates the MAPK signaling cascades. Upon MAPK signaling activation, the levels of FOXO1 and p21 are reduced; consequently, the expression and activity of cyclins and CDKs are upregulated. On the other hand, the attenuated WNT antagonist DKK1 and increased expression of STAT3 and KIT are observed upon CD83 overexpression-triggered MAPK activation. The uncropped western blots of Figure 6b,c,f in the Figure S15–17.

Next, we identified the region within CD83 to mediate the interaction between CD83 and TAK1-TAB1 complex. Membrane CD83 protein contained an extracellular domain, a transmembrane domain, and an intracellular domain (residues 167–205) (Figure 6a). We generated a truncation mutant of the CD83 intracellular domain (termed CD83ΔID) with a Flag tag and found that the deletion of the intracellular domain significantly blocked the interaction of CD83 with TAK1 and TAB1 in a Co-IP assay (Figure 6b). As expected, SKOV3 cells transfected with CD83ΔID expressed a lower level of p-TAK1, p-MEK1/2, and p-ERK1/2 as compared with those in the wild-type CD83-transfected SKOV3 cells (Figure 6c). Importantly, CD83-mediated stimulation of cell proliferation and spheroid formation was significantly inhibited in CD83ΔID-transfected SKOV3 cells (Figure 6d,e). Moreover, the deletion of the intracellular domain interrupted the stimulations of CDK2, CCNB1, and STAT3, as well as inhibitions of FOXO1, p21, and DKK1 by CD83 (Figure 6f). Taken together, our findings demonstrated that the CD83 intracellular domain was important for its association with TAK1/TAB1 and activation of the MAPK signaling pathway, as well as CD83 function in ovarian cancer cells.

## 3. Discussion

Emerging pieces of evidence indicate the expression of transmembrane CD83 and secretion of soluble CD83 by various tumor cells, including ovarian cancer cells. Our studies suggest that membrane protein CD83 contributes to the fate determination of ovarian cancer cells at several respects, including cell proliferation, colony formation, spheroid formation, migration, invasion, and tumorigenic capacity of nude mice in vivo, ultimately having an impact on patient survival. However, the relevance of the current studies, such as in vitro cell culture experiments and in vivo tumor formation studies using nude mice, to human ovarian cancer will need to be determined by further clinical studies of human samples.

We have shown that CD83 advances the growth proliferation and colony formation ability but limits the migration and invasion potentials of ovarian cancer cells. Furthermore, CD83 significantly promotes the spheroid formation of ovarian cancer cells in vitro and tumorigenic capacity of nude mice in vivo. An integrated multi-omics approach provides a deeper understanding of the functional interactions between mRNA and protein layers as a complex biological system. Using an integrated analysis of proteomic and transcriptomic data, we have observed the positive regulation of proliferation/stemness factors (e.g., cyclins-CDKs and KIT) and negative regulation of MMPs (e.g., MMP1 and MMP7) by CD83 in ovarian cancer cells. The roles of cyclins and their catalytic partners, the CDKs, as core components of the machinery that drives cell cycle progression, are well established [55]. Cancer is characterized by uncontrolled tumor cell proliferation resulting from the aberrant activity of cell cycle proteins. Furthermore, dysregulated CDKs have been linked to cancer progression, and pharmacological CDK inhibition has recently emerged as a novel and promising approach in cancer therapy (reviewed in [56]). Ovarian cancer stem cells (CSCs), also known as cancer-initiating cells, are characterized by cell surface expression of CD24, CD44, CD117 (KIT), SOX2, and NANOG [57,58,59]. Monoclonal antibodies of anti-CSC markers may be a potential strategy for the treatment of human ovarian cancer (reviewed in [60]). Cancer metastasis is facilitated by the remodeling of the extracellular tumor matrix by a family of proteolytic enzymes known as the MMPs [61]. MMP1 and MMP7 are well-known metalloproteinases to facilitate metastasis and peritoneal dissemination in ovarian cancer, and their overexpression is linked to advanced cancer stages and poor prognosis [62,63]. Accordingly, there is positive regulation of above cell cycle/stemness factors and negative regulation of MMPs by CD83, partially explaining why CD83 advances the growth proliferation and spheroid formation but limits invasion of ovarian cancer cells. However, the molecular mechanisms underlying the direct and/or indirect regulation of those key proteins by membrane CD83 need further investigation.

IP/MS is a powerful approach to characterize protein complexes in cells. We provide the first IP/MS study of membrane protein CD83-interacting proteins in cells to identify the association of CD83 with MAP3K members TAK1 and TAB1 (an indispensable functional mediator between TGF-β and TAK1) on the cell membrane. TAK1 and TAB1 are preassociated and phosphorylated on the membrane to trigger the MAPK signaling pathway [52,64]. Although the TAK1 complex activation has been studied extensively, the investigations regarding regulators of TAK1-TAB1 complex in ovarian cancer cells remain elusive. In this study, we suggest that membrane protein CD83 activates MAPK signaling pathway through the interaction with TAK1-TAB1 complex in ovarian cancer cells. p-TAK1, p-MEK1/2, and p-ERK1/2 are activated by the overexpression of full-length CD83 but not by CD83 lacking the intracellular domain. On the other hand, CD83 stabilizes the expression of TAB1 in ovarian cancer cells upon protein degradation.

FOXO1, a member of the FOXO family of transcription factors, is characterized by a forkhead DNA binding domain [65]. FOXO1 is known as a tumor suppressor, and dysregulation of FOXO1 is involved in a variety of tumorigenesis. FOXO1 can induce cell cycle arrest by increasing the expression of p27 and p21 and decreasing the expression of cyclins [8,9]. Several findings have linked the MAPK-ERK1/2 signaling pathway to the regulation of tumor suppressor FOXO1 as follows. Activation of ERK kinases contributes to FOXO1 downregulation in various types of cancer cells [66,67]. Phosphorylation of XBP1u by ERK1/2 is critical for the degradation of FOXO1 by the proteasome [68]. We identify the activated TAK1-MEK1/2-ERK1/2 signaling, increased CDK2/cyclin B1 expression, and reduced FOXO1/p21 levels in CD83-overexpressed ovarian cancer cells, indicating that MAPK-ERK1/2-FOXO1 axis is involved in the activation of ovarian cancer cell proliferation by CD83.

Previous studies have defined the critical role of the STAT3-DKK1 signaling pathway in the generation of CSCs in a variety of tumors, including ovarian cancer [12,13,14]. Our study supports the notion that STAT3-DKK1 signaling maintains ovarian cancer stemness. The increased STAT3 expression and attenuated wingless-type MMTV integration site family (WNT) antagonist DKK1 were observed in CD83-overexpressed ovarian cancer cells. Furthermore, the transcript levels of many WNT antagonists, such as suppressors of WNT ligands (e.g., *TRABD2B*, *NOTUM*), suppressors of WNT receptors (e.g., *SFRP1*, *RNF43*, *DKKL1*, *NKD2*), and suppressors of β-catenin/TCF (e.g., *AXIN2*, *SHISA3*, *APCDD1*, *TLE2*), were increased in CD83-KD SKOV3 cells by our RNA-seq analysis. However, the regulatory mechanisms between CD83/MAPK and STAT3/DKK1 signaling pathways need further investigation.

CD83 is a glycosylated protein existing naturally in membrane-bound and soluble forms. Soluble CD83 (sCD83), the extracellular domain of the membrane-bound CD83, is released by CD83-positive cells [24]. In addition to known immunity regulation [69], it will be interesting to determine the effects of sCD83 on tumor cell behaviors, including cell proliferation, stemness, invasion, and progression, in vivo. We cannot exclude the possibility that the effects of CD83 on the fate determination of ovarian cancer cells identified in this study are partially contributed by sCD83. Moreover, other deletion mutants (e.g., loss of extracellular domain) will be useful to fully explore the interaction between CD83 and TAK1/TAB1 and the possible role of the extracellular domain.

## 4. Materials and Methods

### 4.1. Ethical Approval

This research has been approved by the Ethics and Animal Welfare Committee, College of Life Sciences, Beijing Normal University (CLS-EAW-2019-030).

### 4.2. Cell Culture

The human ovarian serous adenocarcinoma cell lines SKOV3 (ATCC^®^HTB-77^TM^), OVCAR3 (ATCC^®^HTB-161^TM^), and Caov3 (ATCC^®^HTB-75^TM^) were purchased from American Type Culture Collection (ATCC, Richmond, VA, USA). The human ovarian surface epithelial cell line HOSEpiC was obtained from the Cell Bank of the Chinese Academy of Sciences (Shanghai, China). Cells were cultured in McCoy’s 5a medium, RPMI-1640, or DMEM (Invitrogen, Beijing, China) containing 10% fetal bovine serum (Invitrogen), 1% penicillin/streptomycin (Invitrogen) at 37 °C with 5% CO_2_.

### 4.3. Generation of Stable Cell Lines

To generate human CD83 stable overexpression or knockdown ovarian cancer cell lines, LV5-full length CD83 lentivirus (CD83-OV) and triple LV3-CD83-shRNA lentivirus (CD83-KD) (LV3-CD83-258, 5′-GCT CCG AAG ATG TGG ACT TGC-3′; LV3-CD83-486, 5′-GGA CAT ACA GGT GCA CTC TGC-3′; LV3-CD83-679, 5′-GCT ACA GAG TAT CTT CCC AGA-3′) were synthesized by GenePharma (Shanghai, China) and transduced SKOV3, OVCAR3, and Caov3 cell lines. A pool of LV5 (EF-1a/GFP + Puro) and LV3 (pGLVH1/GFP + Puro) lentivirus was served as a negative control (NC) for CD83-OV and CD83-KD. Polybrene (4 μg/mL) was used to increase the lentivirus infection efficiency. Stable infected cell lines (GFP^+^) could be obtained by two rounds of 1 μg/mL puromycin (Beyotime, Shanghai, China) selection.

### 4.4. Cell Proliferation Assay

Cell proliferation assay was examined using the Cell Counting Kit-8 (CCK-8) Assay Kit (Beyotime). Cells were seeded into 96-well cell culture plates for each group and assayed at 24, 48, 72, 96, and 120 h. CCK-8 was added into each well, and the absorbance was read at 450 nm with a microplate spectrophotometer (ThermoFisher Scientific, Jackson, MS, USA).

### 4.5. Colony Formation Assays

Ovarian cancer cells were plated in triplicate at 500 cells per 60 mm dish. After 7 days, the colonies were stained with 0.1% crystal violet solution (Solarbio, Beijing, China) and counted using a light microscope.

### 4.6. Transwell Assays

A 24-well transwell chamber with an 8-μm-pore polyethylene terephthalate (PET) membrane (Corning, NY, USA) was used for migration assay. The lower chamber was filled with 700 μL media containing 20% FBS, and approximately 10^5^ cells in 200 μL medium without FBS were seeded in the top chamber. The cells were allowed to migrate for 20 h. After removal of the non-migrating cells on the upper face of the membrane, the remaining cells were washed, fixed, and stained with 0.1% crystal violet. The number of migrating and invading cells was counted in six fields randomly. For invasion assay, transwell membrane was pretreated with a mixture of 100 μL Matrigel (Corning) and 300 μL McCoy’s 5a medium at 37 °C for 30 min.

### 4.7. Wound-Healing Assay

The ovarian cancer cells were seeded into 12-well plates (Corning) and cultured until 90% confluence. Then, the surface of the cells was scratched with a 200 μL pipette tip. After washing twice using PBS, the cells were incubated with McCoy’s 5a medium. Photographs were taken at 0, 7, and 20 h.

### 4.8. Spheroid Formation Assay

The 1 × 10^4^ ovarian cancer cells were seeded onto 24-well ultra-low attachment plates (Corning) in cancer stem cell culture medium (McCoy’s 5a medium supplemented with 10 ng/mL EGF, 20 ng/mL bFGF, 5 μg/mL insulin, 0.4% BSA, and 2% B27 supplement). Images of the spheroids were obtained, and the number of spheroids was counted under a microscope 7 days after cell seeding.

### 4.9. In Vivo Tumor Formation Assay

Animal experiments were approved by the Institutional Ethics Committee of Beijing Normal University and in accordance with institutional and national guidelines. Eight-week-old female athymic nude mice were purchased from Charles River (Beijing, China) and housed in pathogen-free conditions. To establish the tumors, 1 × 10^6^ CD83-KD, CD83-OV, and NC-treated SKOV3 cells were injected intraperitoneally. Mice were euthanized after two months, and tumor weight, ascetic fluid, number, and locations of tumors were recorded.

### 4.10. RNA Sequencing

Total RNA (1 μg for each sample) was extracted from SKOV3 cells using RNAsimple Total RNA Kit (Tiangen, Beijing, China). Three duplicate samples for each group were included (Figure S5). Then, equal amounts of total RNA were analyzed using the BGISEQ-500 platform, and the sequencing procedure was performed by BGI (Shenzhen, China). DE genes were selected based on *p*-value and fold change. The *p*-value calculation (asymptotic) and multiple testing correction (Benjamini Hochberg FDR) were further applied to acquire gene entities with significant *p*-value < 0.05 and a fold change of at least 2 fold (Table S1). Raw data are deposited in the GEO database (accession ID: GES125011).

### 4.11. Proteomics Analysis

After protein extraction and trypsin digestion, peptides were labeled by Tandem Mass Tag (TMT) reagents. HPLC fractionation and LC-MS/MS, protein database searching, and bioinformatics analysis of DE proteins were performed by BGI (Shenzhen, China). The detailed procedures could be found in Supplementary Methods. DE genes were selected by |fold change| ≥ 1.5 and significance *p*-value < 0.05 (Tables S2–4). The proteome data have been deposited to the ProteomXchange with identifier PXD013433.

### 4.12. Immunofluorescence

Five-micron paraffin-embedded ovarian cancer and para-cancer tissue sections (OriGene, CT565862) were deparaffinized, rehydrated, and subjected to antigen retrieval in 10 mM sodium citrate buffer. Sections were blocked in blocking buffer (goat serum, 0.3% Triton X-100 in PBS) at room temperature for 45 min and then incubated with a rabbit anti-CD83 antibody (Abcam, ab205343; 1:100) overnight at 4 °C. Sections were washed and incubated in anti-rabbit IgG H&L (Alexa Fluor^®^ 488, Abcam; 1:200) for 1 h. Sections were finally stained with DAPI (Beyotime; 1:1000) to label the nuclei.

### 4.13. IP-MS

The human *CD83* gene (NM_004233.4) fused with a 3×Flag tag sequence at its 3′-end was synthesized by PCR-based accurate synthesis and inserted into a pCDH-MCS-EF1-copGFP-T2A-Puro lentiviral vector (System Biosciences, Los Angeles, CA, USA) between EcoRI and NotI restriction sites. Recombinant lentivirus was prepared, and the stable cell lines expressing CD83-3×Flag were further generated. The CD83-3×Flag-expressing cells and control cells were lysed and immunoprecipitated with anti-Flag magnetic beads (Sigma-Aldrich, San Diego, CA, USA). The purified protein complexes were separated by SDS-PAGE, stained with Coomassie blue staining, and the specific bands in CD83-3×Flag pulled-down sample were excised for the following in-gel trypsin digestion. LC-MS/MS analysis was performed on a Nano Acquity UPLC system (Waters Corporation, Boston, MA, USA) connected to a Q-Exactive HF (ThermoFisher Scientific). Data were searched against the Swiss-Prot Human database, and protein, sequenced at least two peptides, was considered as reliable identification (Table S5).

### 4.14. Western Blot

Proteins were electrophoresed in 10% SDS-PAGE gels and transferred to nitrocellulose membranes. The blots were blocked in 5% milk and incubated overnight at 4 °C with the primary antibodies, followed by incubation with anti-rabbit or mouse IgG H&L (HRP) at 1/10,000 dilution for 1 h. The specific signals and the corresponding band intensities were evaluated using the Odyssey Infrared Imaging System (Odyssey, Berlin, Germany). The protein level was normalized against β-actin. The antibodies used in the present study are listed in Table S6.

### 4.15. ELISA

MMP1 and MMP7 concentrations from cell lysate and cell culture supernatant were measured using MMP1 ELISA Kit (Abcam, Cambridge, MA, USA) and MMP7 ELISA Kit (CUSABIO, Wuhan, China), respectively, following the manufacturer’s instructions. Concentrations (pg/mL) were determined by absorbance measurements at 450 nm against a standard curve in a competitive assay using an ELISA reader (DeTie, Nanjing, China).

### 4.16. Co-IP

The CD83-3×Flag, CD83 ΔID-3×Flag (CD83 mutant without the intracellular domain), or control cells were lysed and immunoprecipitated with anti-Flag magnetic beads (Sigma-Aldrich, St. Louis., MO, USA). Both the input and IP samples were analyzed by Western blotting using antibodies, including Flag, TAK1, TAB1, and β-actin. The antibodies are listed in Table S6.

### 4.17. Statistics

Data were compared for statistical significance using GraphPad Prism version 5.01 (Graph Pad Software, San Diego, CA, USA). Student’s *t*-test (for two groups) was used for the statistical analyses. The data were presented as the mean±SEM, and differences were considered statistically significant at * *p* < 0.05, ** *p* < 0.01.

## 5. Conclusions

In summary, the in vitro and in vivo data presented here identify the critical roles of membrane protein CD83 in ovarian cancer cells. Our results further show the mechanisms underlying the stimulation of proliferation and stemness of ovarian cancer cells by CD83, involving interaction with MAP3K7 and activation of MAPK signaling pathway, as well as downstream FOXO1/p21/CDK2/Cyclin B1 and STAT3/DKK1 cascades.

## Supplementary Materials

The following are available online at https://www.mdpi.com/2072-6694/12/8/2269/s1, The online version of this article contains supplementary material, which is available to authorized users. Raw data of RNA sequencing is deposited in the GEO database (accession ID: GES125011). The proteome data have been deposited to the ProteomXchange (accession ID: PXD013433). IP-MS data are listed in Supplementary Table S5. Figure S1: Expression profile of CD83 and CD83 highly-associated genes in human ovarian cancers, Figure S2: Establishment of CD83 stable overexpression and knockdown sublines of ovarian cancer cells, Figure S3: Colony formation, spheroid formation and invasive abilities of ovarian cancer cells, Figure S4: CD83 does not affect the apoptosis of ovarian cancer cells as indicated by TUNEL (terminal dexynucleotidyl transferase (TdT)-mediated dUTP nick end labeling) staining, Figure S5: Protein levels of KIT, MMP1, and MMP7 in ovarian cancer cells, Figure S6: Sample quality of transcriptome and proteome, Figure S7: Expression of MMPs, ADAMs, ADAMTSs, and their inhibitors (TIMPs), Figure S8-S18: The uncropped western blots, Table S1: Transcriptome analysis (KD>NC>OV & OV>NC>KD). The transcriptome data have been deposited to GEO database with the identifier GSE125011, Table S2: Integrated analyses of proteome and transcriptome (KD-vs.-NC), Table S3: Integrated analyses of proteome and transcriptome (OV-vs.-NC), Table S4: Integrated analyses of proteome and transcriptome (OV-vs.-KD), Table S5: Putative CD83 interactors identified by CD83 IP-MS, Table S6: The information regarding the antibodies used in this study.
