# Supplementary material for: CD83, a Novel MAPK Signaling Pathway Interactor, Determines Ovarian Cancer Cell Fate
Source: Cancers (Basel). 2020 Aug 13;12(8):2269. doi: 10.3390/cancers12082269 (PMC7465057; doi:10.3390/cancers12082269)
Supplement: Supplementary file 1 [file cancers-12-02269-s001.pdf]

Supplementary Materials: CD83, A Novel MAPK Signalling Pathway Interactor, Determines Ovarian Cancer Cell Fate

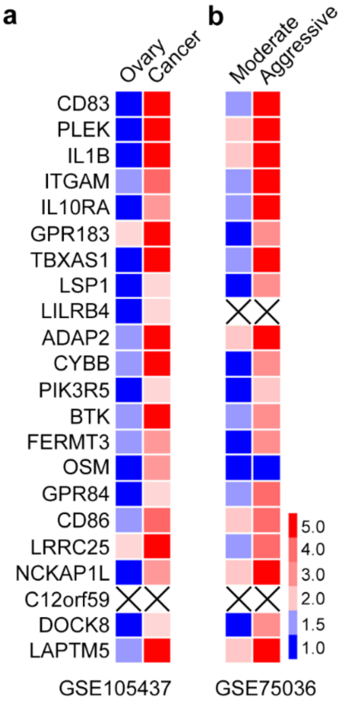

Figure S1. Expression profile of *CD83* and *CD83* highly-associated genes in human ovarian cancers. (**a**) The levels of *CD83* and *CD83* highly-associated genes (Person correlation >0.5, $p < 0.01$) within ovarian cancer transcriptome were higher (logFC >2, $p < 0.01$) in EOC ($n = 10$) than that in normal ovary tissues ($n = 5$) as revealed by GSE105437. (**b**)Among EOC subpopulations, transcripts of CD83 and *CD83* highly-associated genes were significantly upregulated (logFC >2, $p < 0.01$) in EpCAM$^+$CD45$^+$ highly aggressive, drug-resistant, and ovarian cancer stem cell-containing tumor cells compared to EpCAM$^+$ cells as revealed by GSE75036.

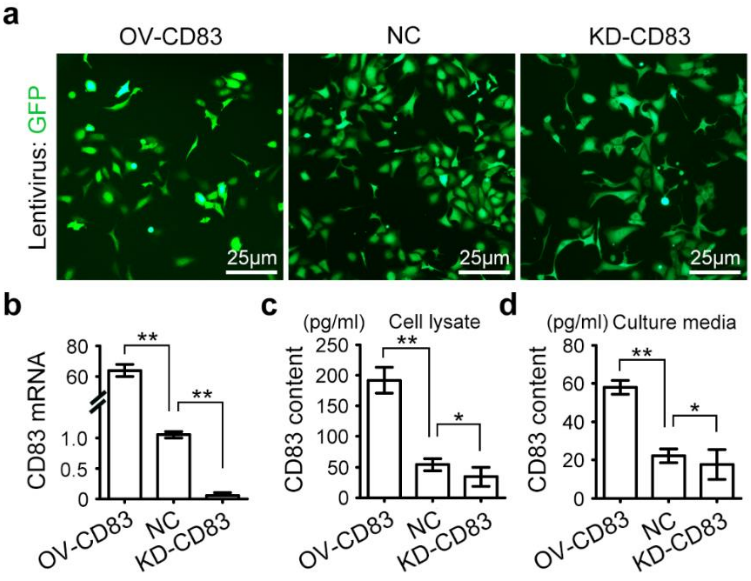

Figure S2. Establishment of CD83 stable overexpression and knockdown sublines of ovarian cancer cells. (**a**) After the infection of full length CD83 lentivirus (OV-CD83), CD83-specific lentiviral shRNA (KD-CD83), or negative control lentivirus (NC), GFP-positive stable transfected cell lines were generated by two rounds of 1 μg/ml puromycin (ST551, Beyotime Biotechnology, Shanghai, China) selection. Scale bar, 25 μm. (**b**) Relative CD83 mRNA level in OV-CD83, NC, and KD-CD83 ovarian cancer cells using qRT-PCR assay. (**c,d**) Enzyme linked immunosorbent assay (ELISA) assay was utilized to examine the protein content (pg/ml) of CD83 in cell lysate (**c**) and culture supernatant (d) of CD83-OV, NC and CD83-KD ovarian cancer cells. The data in (**b**–**d**) were presented as the mean±SEM, Student's *t* test; **p* < 0.05, ***p* < 0.01.

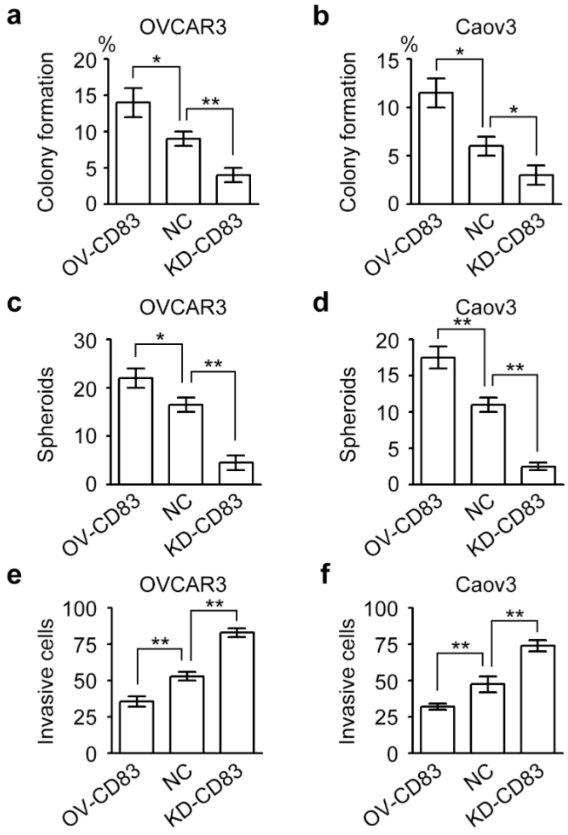

Figure S3. Colony formation, spheroid formation and invasive abilities of ovarian cancer cells. (**a,b**) Colony formation of cells (500 cells per 60 mm dish) is significantly promoted by enforced expression of CD83 in OVCAR3 or Caov3 ovarian cancer cells. (**c,d**) Number of spheroids derived from OVCAR3 or Caov3 ovarian cancer cells which are cultured onto ultra-low attachment plates in cancer stem cell culture medium. (**e,f**) Quantification of invasive OVCAR3 or Caov3 ovarian cancer cells cells in OV-CD83, NC, and KD-CD83 transwells using matrigel invasion assay.

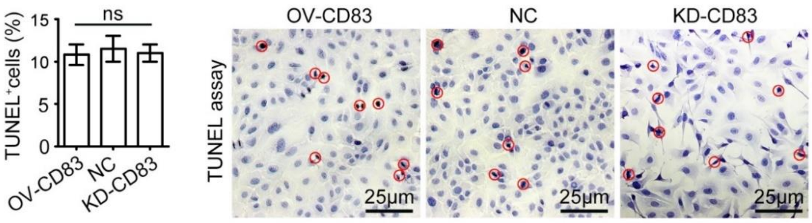

Figure S4. CD83 does not affect the apoptosis of ovarian cancer cells as indicated by TUNEL (terminal dexynucleotidyl transferase (TdT)-mediated dUTP nick end labeling) staining. All data are presented as the mean±SEM of triplicate experiments. Student's *t* test. ns means not significant.

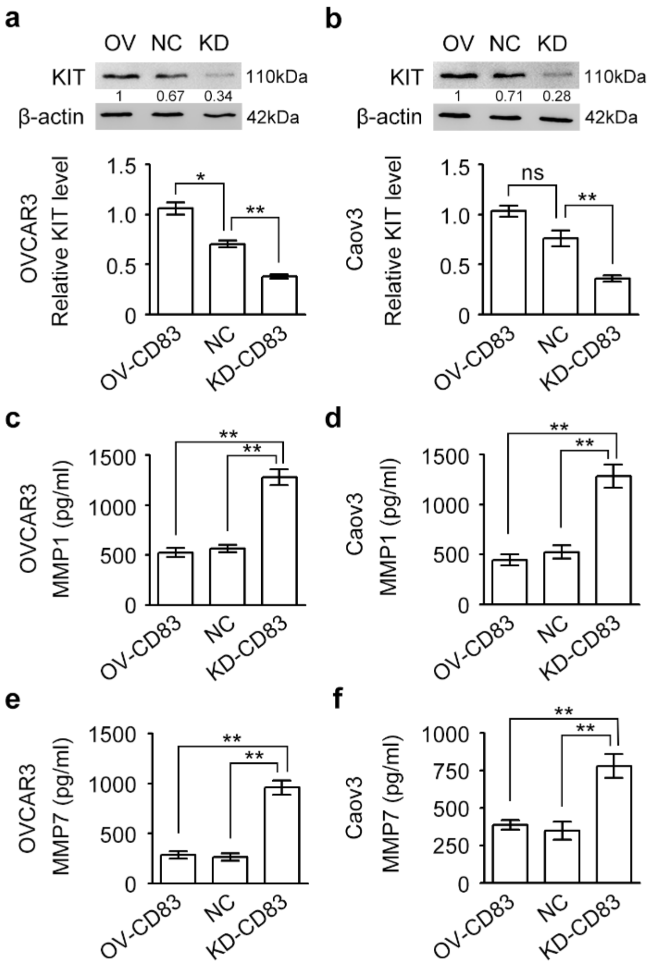

Figure S5. Protein levels of KIT, MMP1, and MMP7 in ovarian cancer cells. (**a,b**) Expression of stemness factor KIT in OVCAR3 and Caov3 ovarian cancer cells identified by Western blotting. Content of MMP1 (**c,d**) and MMP7 (**e,f**) in cell lysate and culture media by ELISA assay.–The data were presented as the mean ± SEM, Student's *t* test; *$p < 0.05$, **$p < 0.01$, ns means not significant. No significant difference was observed between OV and NC in **c**–**f**.

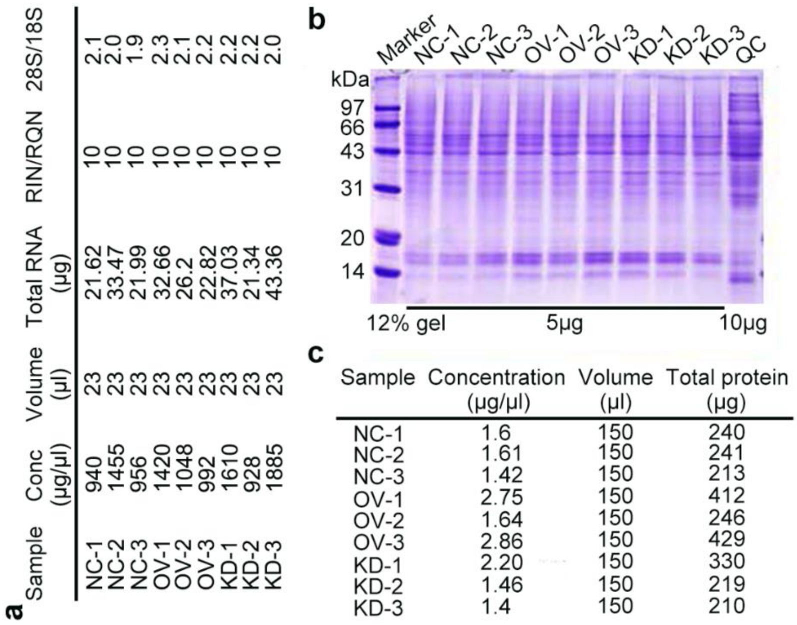

Figure S6. Sample quality of transcriptome and proteome. (**a**) Total RNA content (>20 µg), RNA integrity number (RIN) value (>8), and 28S/18S value (1.9~2.3) of NC, OV-CD83, and KD-CD83 SKOV3 cells (3 samples for each group). (**b**) Coomassie brilliant blue staining of 5 µg samples in 12% SDS-PAGE gel. (**c**) Total protein content for each sample (>200 µg).

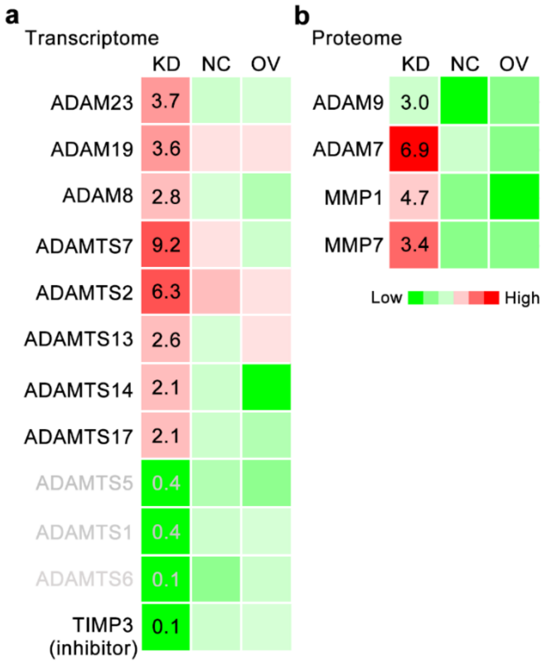

Figure S7. Expression of MMPs, ADAMs, ADAMTSs, and their inhibitors (TIMPs). (**a**) Transcriptome shows the relative levels (FPKM values) of ADAMs (*ADAM8*, *19*, *23*), ADAMTSs (*ADAMTS1*, *2*, *5*, *6*, *7*, *13*, *14*, *17*), and *TIMP3* among KD-CD83, NC, and OV-CD83 SKOV3 cells. (**b**) Protein levels of ADAMs (AMAM7, 9) and MMPs (MMP1, 7) among KD-CD83, NC, and OV-CD83 samples as revealed by proteomics. MMP, matrix metalloproteinase; ADAM, ADAM metallopeptidase; ADAMTS, ADAM metallopeptidase with thrombospondin type 1; TIMP, TIMP metallopeptidase inhibitor.

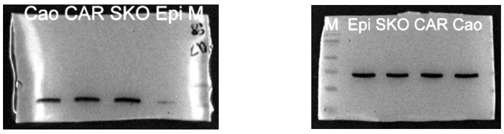

CD83 β-actin

Figure S8. The uncropped western blot figures of Figure 1a

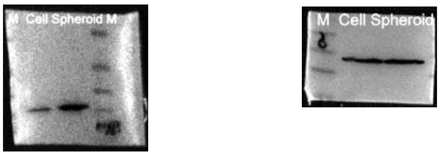

CD83 β-actin

Figure S9. The uncropped western blot figures of Figure 1b

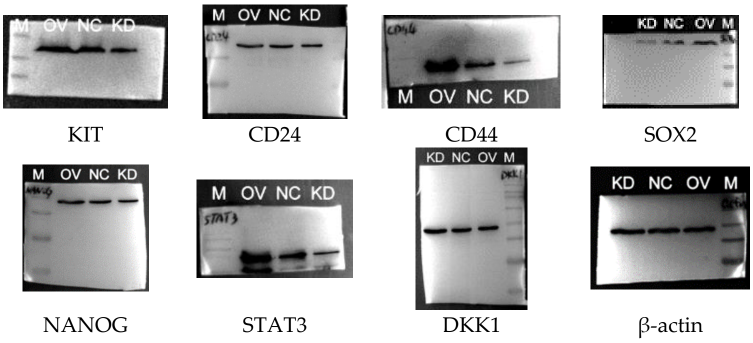

Figure S10. The uncropped western blot figures of Figure 2d

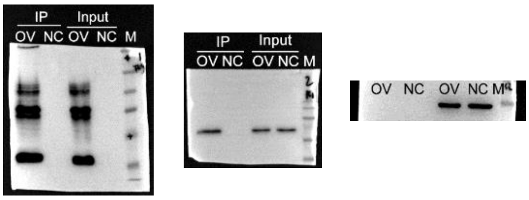

TAB1 TAK1 β-actin

Figure S11. The uncropped western blot figures of Figure 5c

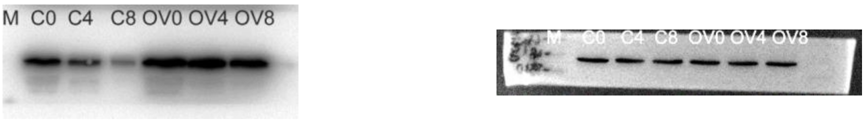

TAB1 β-actin

Figure S12. The uncropped western blot figures of Figure 5d

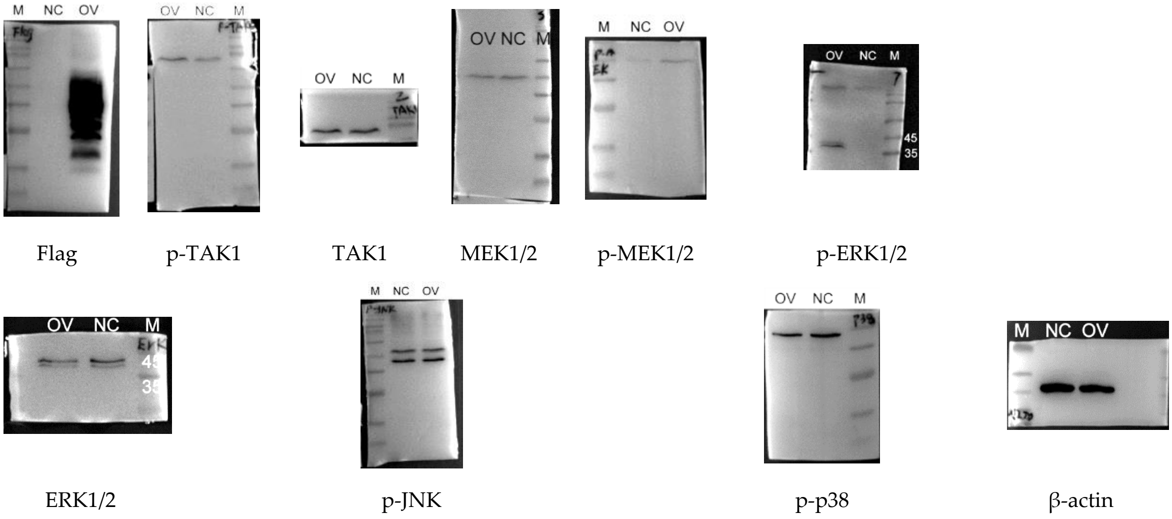

Figure S13. The uncropped western blot figures of Figure 5e

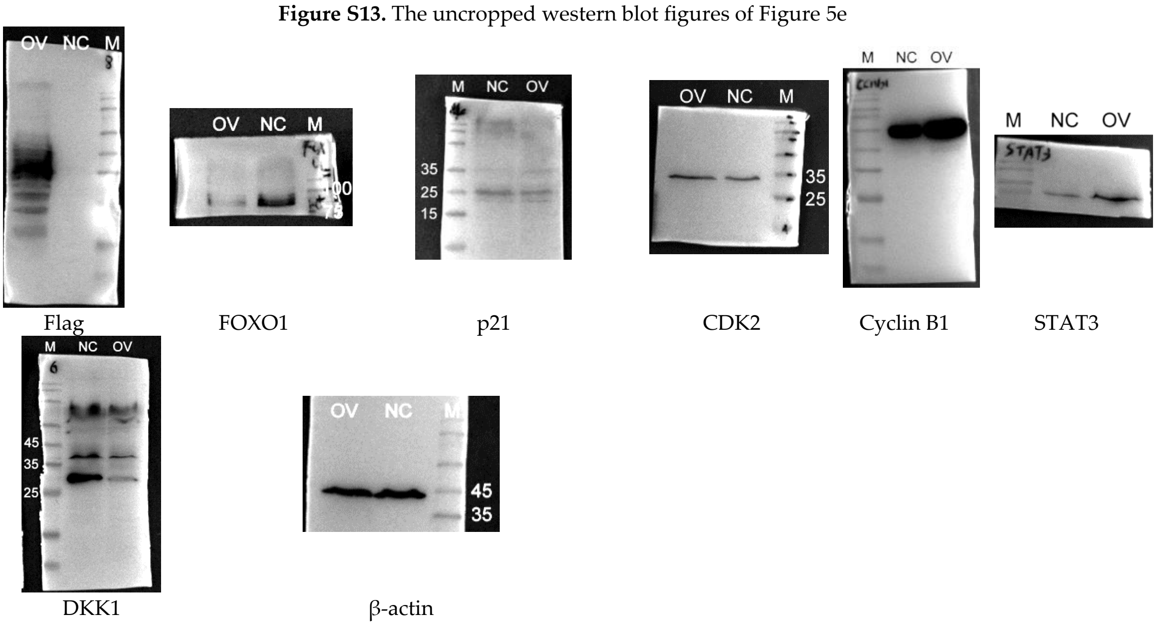

Figure S14. The uncropped western blot figures of Figure 5g.

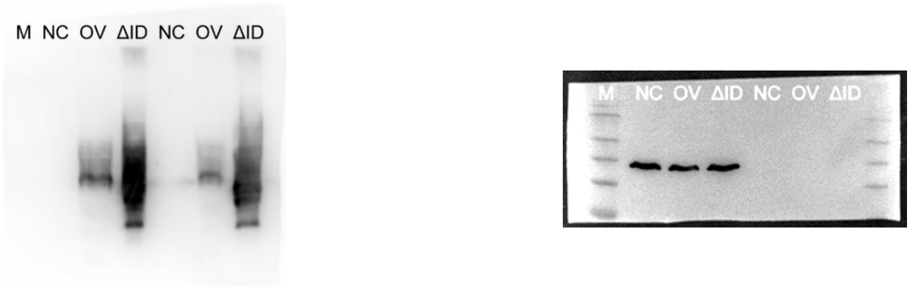

Flag β-actin

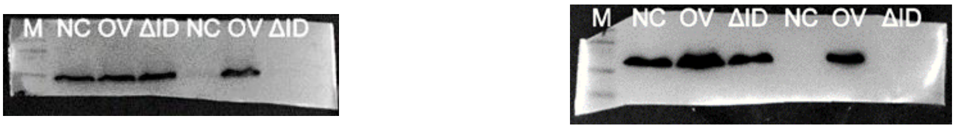

TAK1 TAB1

Figure S15. The uncropped western blot figures of Figure 6b

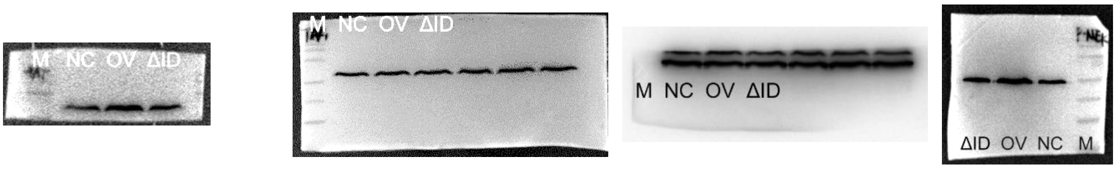

p-TAK1 TAK1 MEK1/2 p-MEK1/2

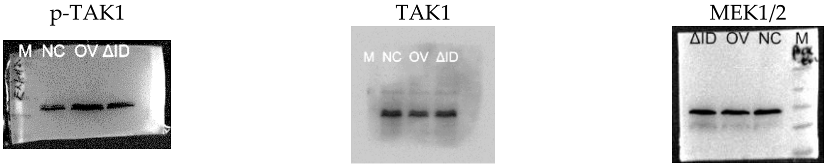

p-ERK1/2 ERK1/2 β-actin

Figure S16. The uncropped western blot figures of Figure 6c

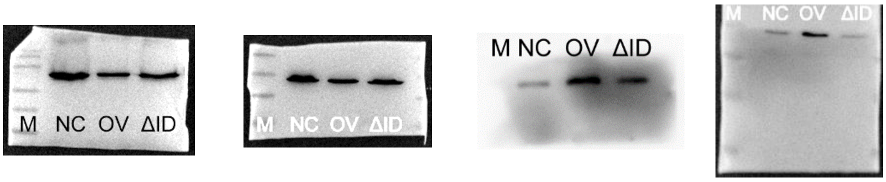

FOXO1 p21 Cyclin B1 CDK2

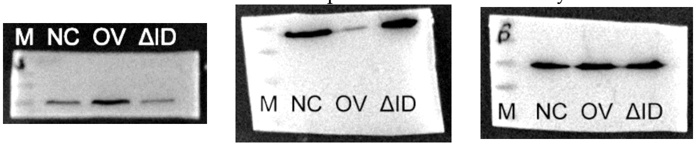

STAT3 DKK1 β-actin

Figure S17. The uncropped western blot figures of Figure 6f

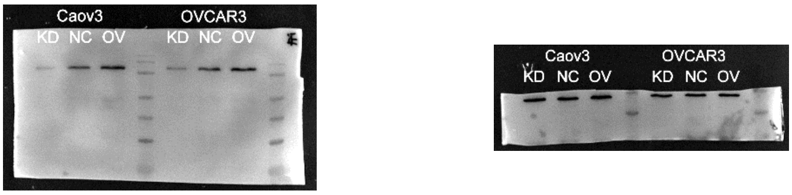

KIT (OVCAR3) KIT (Caov3) β-actin (OVCAR3) β-actin (Caov3)

Figure S18. The uncropped western blot figures of Figure S5

Table S1. Transcriptome analysis (KD>NC>OV & OV>NC>KD). The transcriptome data have been deposited to GEO database with the identifier GSE125011.

Symbol	log(KD/NC)	log2(OV/NC)	Official Full Name
RASGEF1A	1.402625027	−1.014121357	RasGEF domain family member 1A
ZNF423	2.620363686	−1.018897778	zinc finger protein 423
HAP1	1.402367411	−1.020399803	huntingtin associated protein 1
SBK1	1.177738032	−1.043835929	SH3 domain binding kinase 1
COL8A2	1.231867974	−1.06055693	collagen type VIII alpha 2 chain
CLDN7	1.002682698	−1.121192728	claudin 7
ADAMTS14	1.07986488	−1.138517698	ADAM metallopeptidase with thrombospondin type 1 motif 14
MMP10	1.228157265	−1.141691557	matrix metallopeptidase 10
TM7SF2	1.051229841	−1.152836416	transmembrane 7 superfamily member 2
IFITM1	1.915422129	−1.156370708	interferon induced transmembrane protein 1
WNT6	2.462942638	−1.168644898	Wnt family member 6
EFNB3	2.623058731	−1.203391708	ephrin B3
CYP26A1	1.061942972	−1.284295777	cytochrome P450 family 26 subfamily A member 1
LARGE2	1.891408596	−1.324566459	LARGE xylosyl- and glucuronyltransferase 2
NOX4	1.266203127	−1.397427336	NADPH oxidase 4
DHRS3	2.292240592	−1.413006257	dehydrogenase/reductase 3
GIP	1.061942972	−1.523291171	gastric inhibitory polypeptide
TMEM63C	1.288451502	−1.529487437	transmembrane protein 63C
MPPED2	1.281004197	−1.674884285	metallophosphoesterase domain containing 2
CA11	1.354724722	−1.680312339	carbonic anhydrase 11
GDF15	1.375483282	−1.689301123	growth differentiation factor 15
OAS1	1.40433517	−1.692986169	2'-5'-oligoadenylate synthetase 1
ABO	2.646905473	−3.673533807	ABO, alpha 1-3-N-acetylgalactosaminyltransferase and alpha 1-3-galactosyltransferase
BAALC	1.993555997	−4.727981591	BAALC, MAP3K1 and KLF4 binding
FCGBP	2.680852805	−4.780449011	Fc fragment of IgG binding protein
MTPN	1.646950284	−9.303786928	myotrophin
KRT34	−3.491417531	2.302126218	keratin 34
CPED1	−3.053534245	1.718783616	cadherin like and PC-esterase domain containing 1
FLRT2	−2.431366333	1.565698231	fibronectin leucine rich transmembrane protein 2
FGF1	−1.938057028	1.422491048	fibroblast growth factor 1
INHBA	−1.171070565	1.352328922	inhibin subunit beta A
BNC1	−1.247385086	1.331007009	basonuclin

Table S2. Integrated analyses of proteome and transcriptome (KD-vs.-NC).

Symbol	Proteome (Ratio)	Transcriptome (Ratio)	Official Full Name
ROBO1	0.42	0.43	roundabout homolog 1 isoform X9
ARFGAP2	0.51	0.36	ADP-ribosylation factor GTPase-activating protein 2 isoform X8
PDE1C	0.56	0.40	calcium/calmodulin-dependent 3', 5'-cyclic nucleotide phosphodiesterase 1C isoform 1
FBN2	0.54	0.20	fibrillin-2 isoform X1
PTPRR	0.57	0.16	receptor-type tyrosine-protein phosphatase R isoform 2
WBP2	0.66	0.32	WW domain-binding protein 2 isoform 1
PKP2	0.55	0.11	plakophilin-2 isoform 2a
PCDH9	0.41	0.19	protocadherin-9 isoform X4
PCBD1	0.61	0.31	pterin-4-alpha-carbinolamine dehydratase isoform 1
ZFHX4	0.64	0.34	zinc finger homeobox protein 4 isoform X4
BABAM1	0.60	0.50	BRISC and BRCA1-A complex member 1 isoform 1
AMPH	0.57	0.23	amphiphysin isoform X6
COL1A2	0.57	0.36	collagen alpha-2(I) chain precursor
COL1A1	0.59	0.39	collagen alpha-1(I) chain preproprotein
KRT5	0.57	0.14	keratin, type II cytoskeletal 5
KRT80	0.64	0.30	keratin, type II cytoskeletal 80 isoform K80.1
CLIC3	0.64	0.37	chloride intracellular channel protein 3
UAP1	0.63	0.35	UDP-N-acetylhexosamine pyrophosphorylase isoform a
ALDH3A1	0.65	0.29	aldehyde dehydrogenase, dimeric NADP-preferring isoform X2
CPA4	0.49	0.39	carboxypeptidase A4 isoform 2 precursor

GFRA1	0.54	0.09	GDNF family receptor alpha-1 isoform b precursor
KRT17	0.54	0.12	keratin, type I cytoskeletal 17
DHCR24	0.58	0.14	delta(24)-sterol reductase precursor
KRT14	0.59	0.11	keratin, type I cytoskeletal 14
DCAF12L1	0.61	0.29	DDB1- and CUL4-associated factor 12-like protein 1
PDZK1	0.64	0.07	Na(+)/H(+) exchange regulatory cofactor NHE-RF3 isoform X4
SPDYE3	0.65	0.49	speedy protein E3
TSEN15	0.66	0.40	tRNA-splicing endonuclease subunit Sen15 isoform 1
SFXN2	0.66	0.50	sideroflexin-2 isoform X4
SORBS1	3.53	4.59	sorbin and SH3 domain-containing protein 1 isoform 1
SYNGR3	3.28	3.10	synaptogyrin-3
GJA1	3.26	2.51	gap junction alpha-1 protein
DCHS1	2.92	3.86	protocadherin-16 precursor
MCFD2	2.41	2.07	multiple coagulation factor deficiency protein 2 isoform B
MMP1	2.24	12.91	interstitial collagenase isoform 1 preproprotein
TNFSF10	2.03	4.69	tumor necrosis factor ligand superfamily member 10
CD40	1.97	2.30	tumor necrosis factor receptor superfamily member 5 X5
TM6SF2	1.94	2.14	transmembrane 6 superfamily member 2
IFITM3	1.90	2.23	interferon-induced transmembrane protein 3
LRP12	1.89	3.03	low-density lipoprotein receptor-related protein 12 isoform b precursor
CACNA2D2	1.88	7.46	voltage-dependent calcium channel subunit alpha-2/delta-2
MMP7	1.76	27.09	matrilysin preproprotein
SYNGR1	1.75	2.65	synaptogyrin-1 isoform 1b
REPS2	1.72	10.06	ralBP1-associated Eps domain-containing protein 2 X12
TFPI2	1.68	3.05	tissue factor pathway inhibitor 2 isoform 2 precursor
SLC38A2	1.66	2.25	sodium-coupled neutral amino acid transporter 2 isoform 1
MDGA1	1.66	2.43	MAM domain-containing glycosylphosphatidylinositol anchor protein 1 preproprotein
AATK	1.65	3.89	serine/threonine-protein kinase LMTK1 isoform X7
SLC39A10	1.65	2.07	zinc transporter ZIP10 isoform X3
FAM174B	1.65	2.97	membrane protein FAM174B precursor
GALNT16	1.63	3.29	polypeptide N-acetylgalactosaminyltransferase 16 isoform X2
ALDH2	1.63	2.41	aldehyde dehydrogenase, mitochondrial isoform 1 precursor
ITGB3	1.62	4.44	integrin beta-3 precursor
GOLGA6C	1.61	2.45	golgin subfamily A member 6C isoform X1
TMEM245	1.61	2.39	transmembrane protein 245 isoform X5
IGFBP7	1.61	2.20	insulin-like growth factor-binding protein 7 isoform 2
VSNL1	1.60	6.50	visinin-like protein 1 isoform 1
AIF1L	1.59	3.12	allograft inflammatory factor 1-like isoform 1
NRP2	1.58	3.41	neuropilin-2 isoform X2
HECW2	1.57	2.33	E3 ubiquitin-protein ligase HECW2 isoform 2
NT5E	1.54	8.63	5'-nucleotidase isoform 2 preproprotein
TMEM65	1.54	2.39	transmembrane protein 65
GNG2	1.53	5.43	guanine nucleotide-binding protein G(I)/G(S)/G(O) subunit gamma-2 isoform X2
RAB31	1.52	3.63	ras-related protein Rab-31
ANTXR2	1.51	3.63	anthrax toxin receptor 2 isoform 3

Table S3. Integrated analyses of proteome and transcriptome (OV-vs.-NC).

Symbol	Proteome (Ratio)	Transcriptome (Ratio)	Official Full Name
RSPH4A	2.26	4.86	radial spoke head protein 4 homolog A isoform X1
CD83	1.73	12.21	CD83 antigen isoform c

Table S4. Integrated analyses of proteome and transcriptome (OV-vs.-KD).

Symbol	Proteome (Ratio)	Transcriptome (Ratio)	Official Full Name
RSPH4A	3.63	2.10	radial spoke head protein 4 homolog A isoform X1
ROBO1	3.24	2.35	roundabout homolog 1 isoform X9
GFRA1	2.52	12.17	GDNF family receptor alpha-1 isoform b precursor
FKBP5	2.28	2.92	peptidyl-prolyl cis-trans isomerase FKBP5 isoform 1

PCDH9	2.11	5.23	protocadherin-9 isoform X4
SMTN	2.10	2.13	smoothelin isoform X13
SPANXB1	2.08	9.69	sperm protein associated with the nucleus on the X chromosome B1
AMPH	1.99	3.89	amphiphysin isoform X6
COL1A1	1.93	3.36	collagen alpha-1(I) chain preproprotein
PKP2	1.89	8.83	plakophilin-2 isoform 2a
WBP2	1.85	2.09	WW domain-binding protein 2 isoform 1
HMGA1	1.81	3.34	high mobility group protein HMG-I/HMG-Y isoform b
PDZK1	1.80	13.46	Na(+)/H(+) exchange regulatory cofactor NHE-RF3 isoform
CD83	1.79	147.64	CD83 antigen isoform c
TAGLN	1.77	3.71	transgelin
KRT5	1.76	5.19	keratin, type II cytoskeletal 5
COL1A2	1.74	2.61	collagen alpha-2(I) chain precursor
BCCIP	1.72	2.04	BRCA2 and CDKN1A-interacting protein isoform BCCIPalpha
CXADR	1.68	3.03	coxsackievirus and adenovirus receptor isoform X4
FBN2	1.65	5.32	fibrillin-2 isoform X1
CDCA7L	1.64	2.24	cell division cycle-associated 7-like protein isoform 3
ARID5B	1.64	3.29	AT-rich interactive domain-containing protein 5B isoform 2
S100A2	1.60	2.84	protein S100-A2
DHCR24	1.59	5.97	delta(24)-sterol reductase precursor
KL	1.59	2.35	klotho isoform X1
DCAF12L1	1.58	3.39	DDB1- and CUL4-associated factor 12-like protein 1
KIT	1.57	2.42	mast/stem cell growth factor receptor Kit isoform X6
DYSF	1.56	5.19	dysferlin isoform 3
UAP1	1.55	4.59	UDP-N-acetylhexosamine pyrophosphorylase isoform a
PPME1	1.52	2.18	protein phosphatase methylesterase 1 isoform a
TSEN15	1.52	3.09	tRNA-splicing endonuclease subunit Sen15 isoform 1
SKA3	1.51	2.06	spindle and kinetochore-associated protein 3 isoform X1
GJA1	0.30	0.50	gap junction alpha-1 protein
SYNGR3	0.42	0.20	synaptogyrin-3
MMP1	0.45	0.04	interstitial collagenase isoform 1 preproprotein
TM6SF2	0.46	0.50	transmembrane 6 superfamily member 2
VDAC3	0.46	0.41	voltage-dependent anion-selective channel protein 3 isoform 1
SORBS1	0.47	0.22	sorbin and SH3 domain-containing protein 1 isoform 1
DNAJC25-GNG10	0.48	0.01	DNAJC25-GNG10 protein precursor
EPPK1	0.49	0.01	epiplakin isoform X3
AATK	0.52	0.14	serine/threonine-protein kinase LMTK1 isoform X7
NID1	0.52	0.21	nidogen-1 isoform X1
DCHS1	0.53	0.19	protocadherin-16 precursor
CACNA2D2	0.53	0.11	voltage-dependent calcium channel subunit alpha-2/delta-2
DES	0.55	0.18	desmin
TNFSF10	0.57	0.12	tumor necrosis factor ligand superfamily member 10
CD40	0.58	0.34	tumor necrosis factor receptor superfamily member 5 isoform
IMPA2	0.58	0.31	inositol monophosphatase 2 isoform X1
IFITM3	0.59	0.32	interferon-induced transmembrane protein 3
MMP7	0.59	0.03	matrilysin preproprotein
CACFD1	0.59	0.31	calcium channel flower homolog isoform X5
VSNL1	0.59	0.10	visinin-like protein 1 isoform 1
NRP2	0.61	0.32	neuropilin-2 isoform X2
AIF1L	0.61	0.19	allograft inflammatory factor 1-like isoform 1
TMC7	0.62	0.49	transmembrane channel-like protein 7 isoform e
SLCO4A1	0.62	0.23	solute carrier organic anion transporter family member 4A1
MTDH	0.62	0.49	protein LYRIC isoform X6
CDH13	0.62	0.25	cadherin-13 isoform X2

KCNK1	0.62	0.60	potassium channel subfamily K member 1
TFPI2	0.63	0.44	tissue factor pathway inhibitor 2 isoform 2 precursor
ACSL5	0.63	0.20	long-chain-fatty-acid--CoA ligase 5 isoform b
NT5E	0.63	0.28	5'-nucleotidase isoform 2 preproprotein
HNRNPU	0.63	0.60	heterogeneous nuclear ribonucleoprotein U isoform b
REPS2	0.63	0.10	ralBP1-associated Eps domain-containing protein 2
PLAU	0.64	0.40	urokinase-type plasminogen activator isoform 2
SAA2	0.64	0.13	serum amyloid A-2 protein isoform b precursor
FOXS1	0.64	0.33	forkhead box protein S1
SLC26A11	0.64	0.37	sodium-independent sulfate anion transporter isoform X5
ALDH2	0.64	0.27	aldehyde dehydrogenase, mitochondrial isoform 1 precursor
RDH10	0.65	0.47	retinol dehydrogenase 10
CUBN	0.65	0.23	cubilin isoform X1
C2orf66	0.65	0.38	uncharacterized protein C2orf66 precursor
SYNGR1	0.65	0.38	synaptogyrin-1 isoform 1b
PLSCR4	0.66	0.49	phospholipid scramblase 4 isoform c

Table S5. Putative CD83 interactors identified by CD83 IP-MS

Gene	Score	Coverage	Proteins	Unique Peptides	Peptides	PSMs	AAs	MW[kDa]	calc.pI
CD83	27932.82	68.78	1	18	18	1266	205	23.0	8.18
ATXN10	1450.11	38.32	1	22	22	98	475	53.5	5.25
PLD3	979.18	23.47	1	10	10	53	490	54.7	6.47
DNAAF5	809.36	27.49	1	22	22	50	855	93.5	6.42
BZW2	771.10	38.19	1	20	23	75	419	48.1	6.68
HMOX2	749.78	35.13	1	12	12	62	316	36.0	5.41
HEATR3	671.84	18.97	1	17	17	46	680	74.5	5.11
ATP2B1	658.92	26.80	1	13	28	46	1220	134.6	5.91
TAB1	622.30	35.71	1	13	13	39	504	54.6	5.52
BZW1	595.19	31.50	1	19	22	57	419	48.0	5.92
ATP2B4	563.39	19.90	1	7	22	34	1241	137.8	6.60
ARMC6	527.95	11.58	1	5	5	20	501	54.1	6.24
PLAA	475.42	36.60	1	26	26	45	795	87.1	6.37
TMEM192	448.58	23.99	1	9	9	36	271	30.9	7.99
RTN3	427.00	2.03	1	3	3	32	1032	112.5	4.96
ALG5	381.46	25.62	1	8	8	28	324	36.9	9.28
EXOC8	380.49	13.79	1	10	10	20	725	81.7	5.49
PGRMC1	368.91	40.00	1	8	8	49	195	21.7	4.70
COMMD9	363.52	29.29	1	5	5	18	198	21.8	5.88
HEATR6	363.02	13.38	1	12	12	17	1181	128.7	7.03
TKFC	346.40	22.78	1	10	10	37	575	58.9	7.49
MAP3K7	345.40	16.67	1	9	9	19	606	67.2	7.11
PGRMC2	313.46	22.42	1	5	5	27	223	23.8	4.88
TNPO3	312.69	15.60	1	11	11	20	923	104.1	5.57
AMOT	286.01	9.04	1	10	10	13	1084	118.0	7.64
TNPO2	258.53	6.80	1	2	6	12	897	101.3	5.01

PI4K2A	249.31	22.34	1	9	9	15	479	54.0	8.29
TELO2	245.28	15.05	1	12	12	21	837	91.7	5.76
GLMN	243.59	17.34	1	11	11	23	594	68.2	5.33
CAND2	223.49	10.11	1	8	11	14	1236	135.2	5.68
RAP1GDS1	216.81	17.63	1	8	9	17	607	66.3	5.31
MPZL1	212.29	16.36	1	4	4	15	269	29.1	8.72
SESN2	208.47	11.04	1	4	4	12	480	54.5	5.90
HDAC6	200.33	5.93	1	6	6	7	1215	131.3	5.30
CTNNAL1	177.03	7.22	1	5	5	7	734	81.8	6.64
BRAT1	173.00	16.32	1	11	11	21	821	88.1	5.27
ARFIP2	167.71	35.48	1	10	11	25	341	37.8	6.04
SLC7A1	166.18	6.36	1	3	4	14	629	67.6	5.43
SOS1	161.21	11.70	1	14	14	15	1333	152.4	6.84
AFDN	155.20	3.51	1	5	5	5	1824	206.7	6.47
TMEM33	146.47	14.57	1	3	3	9	247	28.0	9.70
MAP2K4	145.86	21.05	1	6	6	11	399	44.3	8.07
BAX	139.97	23.96	1	4	4	17	192	21.2	5.22
LEMD3	127.02	17.01	1	12	12	20	911	99.9	7.55
MSTO1	126.30	11.58	1	5	5	9	570	61.8	6.11
NDST1	125.80	6.12	1	5	5	8	882	100.8	7.97
TAB2	121.84	8.23	1	4	4	7	693	76.4	8.54
TM9SF3	119.35	3.40	1	2	2	8	589	67.8	7.21
MAPK1	118.52	13.89	1	5	5	8	360	41.4	6.98
CUL3	117.83	7.55	1	5	5	5	768	88.9	8.48
HEATR1	106.99	2.15	1	4	4	5	2144	242.2	6.54
CDC73	105.17	6.97	1	4	4	7	531	60.5	9.61
TMEM43	103.18	21.00	1	7	7	9	400	44.8	8.13
MAP2K2	100.10	8.00	1	3	3	5	400	44.4	6.55
TBK1	95.58	10.15	1	6	6	8	729	83.6	6.79
RSU1	93.19	18.05	1	5	5	5	277	31.5	8.65
STAT1	89.80	13.07	1	8	8	11	750	87.3	6.05
MAGT1	89.00	8.06	1	3	3	5	335	38.0	9.63
TAB3	88.81	11.24	1	5	5	5	712	78.6	8.54
SLC30A1	84.52	14.00	1	5	5	5	507	55.3	6.48
STAT6	77.90	2.95	1	2	2	3	847	94.1	6.23
TSG101	77.42	5.38	1	3	3	3	390	43.9	6.46
GRK2	76.95	12.92	1	9	9	11	689	79.5	7.28
SLC39A14	74.79	6.91	1	3	3	6	492	54.2	5.33
TMED10	74.78	14.61	1	3	3	5	219	25.0	7.44
IGF2R	72.83	2.49	1	6	6	6	2491	274.2	5.94

CNNM3	58.60	8.77	1	6	6	7	707	76.1	6.09
STAT3	52.33	2.99	1	2	2	3	770	88.0	6.30
CSK	49.47	4.67	1	2	2	4	450	50.7	7.06
MAP2K3	47.67	6.34	1	2	2	3	347	39.3	7.43
TMEM214	38.10	3.05	1	2	2	2	689	77.1	9.14
MIGA1	37.52	11.71	1	5	5	5	632	71.0	5.63
CD2AP	33.53	7.82	1	4	4	4	639	71.4	6.40
AAMP	32.90	5.53	1	2	2	2	434	46.7	4.42

Table S6. The information regarding the antibodies used in this study.

Antibody	Application	Company	Catalog	Host species
CD83	Western blot<br>Immunofluorescence	Abcam	ab205343	Rabbit
KIT	Western blot	Abcam	ab32363	Rabbit
CD24	Western blot	Abcam	ab64064	Rabbit
CD44	Western blot	Abcam	ab189524	Rabbit
SOX2	Western blot	Abcam	ab92494	Rabbit
NANOG	Western blot	Abcam	ab109250	Rabbit
STAT3	Western blot	Abcam	ab68153	Rabbit
DKK1	Western blot	Abcam	ab109416	Rabbit
Flag	Western blot	Sigma-Aldrich	F1804	Mouse
TAK1	Western blot	Abcam	ab109526	Rabbit
TAB1	Western blot	Abcam	ab76412	Rabbit
p-TAK1	Western blot	Abcam	ab109404	Rabbit
MEK1/2	Western blot	Cell Signaling Technology	#8727T	Rabbit
p-MEK1/2	Western blot	Cell Signaling Technology	#9154T	Rabbit
p-JNK	Western blot	Abcam	ab124956	Rabbit
p-p38	Western blot	Abcam	ab195049	Rabbit
FOXO1	Western blot	Cell Signaling Technology	#2880	Rabbit
p21	Western blot	BD Biosciences	554228	Rabbit
CDK2	Western blot	Abcam	ab32147	Rabbit
Cyclin B1	Western blot	Abcam	ab32053	Rabbit
β-actin	Western blot	Sigma-Aldrich	A2228	Mouse
Anti-Rabbit IgG H&L (HRP)	Western blot	Abcam	ab205718	Goat
Anti-Mouse IgG H&L (HRP)	Western blot	Abcam	ab205719	Goat
Anti-Rabbit IgG H&L (Alexa Fluor® 488)	Immunofluorescence	Abcam	ab205719	Goat

Supplementary Methods

1. Quantitative RT-PCR

Total RNA was extracted from the CD83-KD, CD83-OV, and NC-treated SKOV3 cells using RNAsimple Total RNA Kit (Tiangen, Beijing, China). First-strand cDNA was synthetized. qRT-PCR was performed with the CFX96 real-time qPCR detection system (Bio-Rad, CA, USA), using the SYBR Green qPCR kit (TransGen Biotech, Beijing, China). Cycle threshold (CT) value was normalized to the value of human *GAPDH*. Primer sequences were obtained from online PrimerBank. Forward primer of *CD83*: 5′-AAG GGG CAA AAT GGT TCT TTC G-3′ and reverse primer of *CD83*: 5′-GCA CCT GTA TGT CCC CGA G-3′; Forward primer of *GAPDH*: 5′-CTG GGC TAC ACT GAG CAC C-3′ and reverse primer of *GAPDH*: 5′- AAG TGG TCG TTG AGG GCA ATG-3′.

2. TUNEL staining

TUNEL assay was performed using the Colorimetric TUNEL Apoptosis Assay Kit (Beyotime) in accordance with the manufacturer's protocol. The number of TUNEL-positive cells was counted in six fields randomly, and the apoptosis index for each field was calculated as the percent of positive cells relative to the total cells.

3. Proteomics analysis

Sample were extracted with Lysis buffer 3 (8 M Urea, 40 m M Tris-HCl or TEAB, pH 8.5) containing 1 mM PMSF and 2 mM EDTA (final concentration). With placing on ice for 5 min, 10 mM DTT (final concentration) was added to the samples. The suspension was sonicated at 200 W for 1 min and then centrifuged at 4°C, 25 000g for 20 min. The supernatant was incubated at 56°C for 1 h. Subsequently, after cooling to room temperature, the sample was incubated with 55 mM IAM (final concentration) for 45 min in the dark room for alkylation. The supernatant containing proteins was quantified by Bradford after centrifuge with 25000g x 20min at 4°C. SDS-PAGE and Coomassie Blue were used to determine the quality of protein extraction. The protein solution (100 μg) with 8 M urea was diluted 4 times with 100 mM TEAB. Trypsin Gold (Promega, Madison, WI, USA) was used to digest the proteins with the ratio of protein:trypsin =40:1 at 37°C overnight. After trypsin digestion, peptides were desalted with a Strata X C18 column (Phenomenex) and vacuum-dried according to the manufacturer's protocol. The peptides were dissolved in 25 μl 0.2 M TEAB with vortexing. After the IBT labeling reagents were recovered to ambient temperature, they were dissolved in 80ul Isopropanol with vortexing, and then transferred and combined with proper samples. Peptide labeling was performed by IBT Reagent Kit according to the manufacturer's protocol. The labeled peptides with different reagents were combined and vacuum-dried for further use. The peptides were separated on a Shimadzu LC-20AB HPLC Pump system coupled with a high pH RP column. The peptides were reconstituted with buffer A (5% ACN, 95% H_2O, adjust pH to 9.8 with ammonia) to 2 ml and loaded onto a column containing 5-μm particles (Phenomenex). The peptides are separated at a flow rate of 1 ml/min with a gradient of 5% buffer B (5% H_2O, 95% ACN, adjust pH to 9.8 with ammonia) for 10 min, 5-35% buffer B for 40min, 35-95% buffer B for 1 min. The system is then maintained in 95% buffer B for 3 min and decreases to 5% within 1 min before equilibrating with 5% buffer B for 10 min. Elution is monitored by measuring absorbance at 214 nm, and fractions are collected every 1 min. The eluted peptides are pooled as 20 fractions and vacuum-dried. Each fraction was resuspended in buffer A (2% ACN, 0.1%FA) and centrifuged at 20,000g for 10 min. The supernatant was loaded on Thermo Scientific™ UltiMate™ 3000 UHPLC system equipped with a trap and an analytical column. The samples were loaded on a trap column at 5 μl/min for 8 min, and then eluted into the homemade nanocapillary C18 column (ID 75 μm x 25 cm, 3 μm particles) at a flow rate 300nl/min. The gradient of buffer B (98% ACN, 0.1% FA) was increased from 5% to 25% in 40 min, and then increased to 35% in 5 min, followed by 2 min linear gradient to 80%, then maintenance at 80% B for 2 min, and finally return to 5% in 1 min and equilibrated for 6 min. The peptides separated from nanoHPLC were subjected into the tandem mass spectrometry Q EXACTIVE HF X (Thermo Fisher Scientific, San Jose, CA) for DDA (data-dependent acquisition) detection by nano-electrospray ionization. The parameters for MS analysis are listed as following: electrospray voltage: 2.0 kV; precursor scan range: 350-1500 m/z at a resolution of 60,000 in Orbitrap; MS/MS fragment scan range: >100 m/z at a resolution of 30,000 in HCD mode; normalized collision energy setting: 30%; dynamic Exclusion time: 30 s; Automatic gain control (AGC) for full MS target and MS2 target: 3e6 and1e5, respectively; The number of MS/MS scans following one MS scan: 20 most abundant precursor ions above a threshold ion count of 20,000.

© 2020 by the authors. Submitted for possible open access publication under the terms and conditions of the Creative Commons Attribution (CC BY) license (http://creativecommons.org/licenses/by/4.0/).
